# Palmitic Acid-BSA enhances Amyloid-β production through GPR40-mediated dual pathways in neuronal cells: Involvement of the Akt/mTOR/HIF-1α and Akt/NF-κB pathways

**DOI:** 10.1038/s41598-017-04175-w

**Published:** 2017-06-28

**Authors:** Jeong Yeon Kim, Hyun Jik Lee, Sei-Jung Lee, Young Hyun Jung, Dae Young Yoo, In Koo Hwang, Je Kyung Seong, Jung Min Ryu, Ho Jae Han

**Affiliations:** 1 Department of Veterinary Physiology, College of Veterinary Medicine, Research Institute for Veterinary Science, Seoul National University, Seoul, 08826 Republic of Korea; 2 BK21 PLUS Program for Creative Veterinary Science Research Center, Seoul National University, Seoul, 08826 Republic of Korea; 3 Department of Pharmaceutical Engineering, Daegu Haany University, Gyeongsan, 38610 Republic of Korea; 4 Department of Anatomy and Cell Biology, College of Veterinary Medicine, Research Institute for Veterinary Science, Seoul National University, Seoul, 08826 Republic of Korea; 5 Korea Mouse Phenotyping Center (KMPC), Seoul, 08826 Republic of Korea; 6 Department of Veterinary Physiology, College of Veterinary Medicine, Chonnam National University, Gwangju, 61186 Korea

## Abstract

The pathophysiological actions of fatty acids (FAs) on Alzheimer’s disease (AD), which are possibly mediated by genomic effects, are widely known; however, their non-genomic actions remain elusive. The aim of this study was to investigate the non-genomic mechanism of extra-cellular palmitic acid (PA) regulating beta-amyloid peptide (Aβ) production, which may provide a link between obesity and the occurrence of AD. In an obese mouse model, a high-fat diet (HFD) significantly increased the expression levels of APP and BACE1 as well as the AD pathology in the mouse brain. We further found that PA conjugated with bovine serum albumin (PA-BSA) increased the expression of APP and BACE1 and the production of Aβ through the G protein-coupled receptor 40 (GPR40) in SK-N-MC cells. PA-BSA coupling with GPR40 significantly induced Akt activation which is required for mTOR/p70S6K1-mediated HIF-1α expression and NF-κB phosphorylation facilitating the transcriptional activity of the *APP* and *BACE1* genes. In addition, silencing of APP and BACE1 expression significantly decreased the production of Aβ in SK-N-MC cells treated with PA-BSA. In conclusion, these results show that extra-cellular PA coupled with GPR40 induces the expression of APP and BACE1 to facilitate Aβ production via the Akt-mTOR-HIF-1α and Akt-NF-κB pathways in SK-N-MC cells.

## Introduction

Alzheimer’s disease (AD), the most common neurodegenerative disease, is characterized by cognitive decline, memory dysfunction and behavioral impairments. The excessive production and aggregation of beta-amyloid peptide (Aβ) and microtubule aggregation induced by abnormal phosphorylation of tau, called a tauopathy, in neuronal cells are considered the primary causes of AD. The aberrant regulation of amyloid precursor protein (APP) and beta-site amyloid precursor protein cleaving enzyme 1 (BACE1) cause the accumulation of Aβ resulting in familial and sporadic AD occurrence^1–5^. Earlier findings have suggested that the regulation of APP processing is important for Aβ production. As a result, this area of research is emerging as a therapeutic target for AD^4, 6^. Thus, studies on the processes leading to Aβ-mediated AD may contribute to uncovering the mechanisms of AD pathogenesis.

Recently, accumulating evidence has shown that the obesity is a potential risk factor for AD^7, 8^. In addition, high fat diet and high cholesterol stimulate amyloidogenic pathways responsible for the pathogenesis of AD^9–11^. These findings provide an important direction for those doing research on neurodegeneration and AD in patients with obesity and metabolic syndrome. An increase in fatty acids (FAs) is one of the main characteristics found in obese patients^12^. Palmitic acid (PA), an abundant saturated FA existing in the human body, is closely linked to metabolic diseases. According to a report by Carine *et al*.^13^, people who are overweight have a higher proportion of PA among all FAs than those who are not overweight regardless of the presence or absence of metabolic syndrome. In neuronal cells, PA is known to induce ER stress and apoptotic cell death and to impair proliferation and alter differentiation^14–16^. There have been several studies investigating the distinct role of genomic and non-genomic actions of PA in AD. Previous researchers showed that PA-BSA treatment stimulates BACE1 expression in astrocyte, and conditioned medium from astrocytes activated by free PA facilitated AD-associated amyloidogenesis through astroglia-mediated oxidative stress^17, 18^. However, the genomic actions mediated by free PA could not directly activate BACE1 expression due to the low uptake and utilization of PA by primary neurons. Therefore, it is necessary to investigate the relationships between the non-genomic actions of PA and AD occurrence. However, there are few studies on the non-genomic actions of PA despite its importance in AD pathogenesis in obese patients. Further understanding of the non-genomic actions of PA will provide insight into the development of effective therapeutic strategies against excess FAs levels that further advance AD progression.

Several studies with animal models have shown an increase in AD-like pathology in the presence of diet-induced obesity and metabolic disturbances^19–21^. Moreover, the literature suggests that dietary components may be important in regulating AD pathology^10, 11, 22^ even in the absence of obesity and metabolic syndrome. A commonly used approach is the use of high-fat diet (HFD) in rodents leading to diet-induced obesity. SK-N-MC cells have been used as a typical *in vitro* cell model to investigate signal transduction in many AD studies^23–26^. This study investigated the effects of a high-fat diet (HFD) on Aβ regulating enzymes in the brain with a C57BL/6 obese mouse model and the non-genomic mechanism of PA in amyloidogenesis in SK-N-MC cells.

## Results

### HFD and PA induce the expressions of APP and BACE1 as well as Aβ production

**Figure 1:**
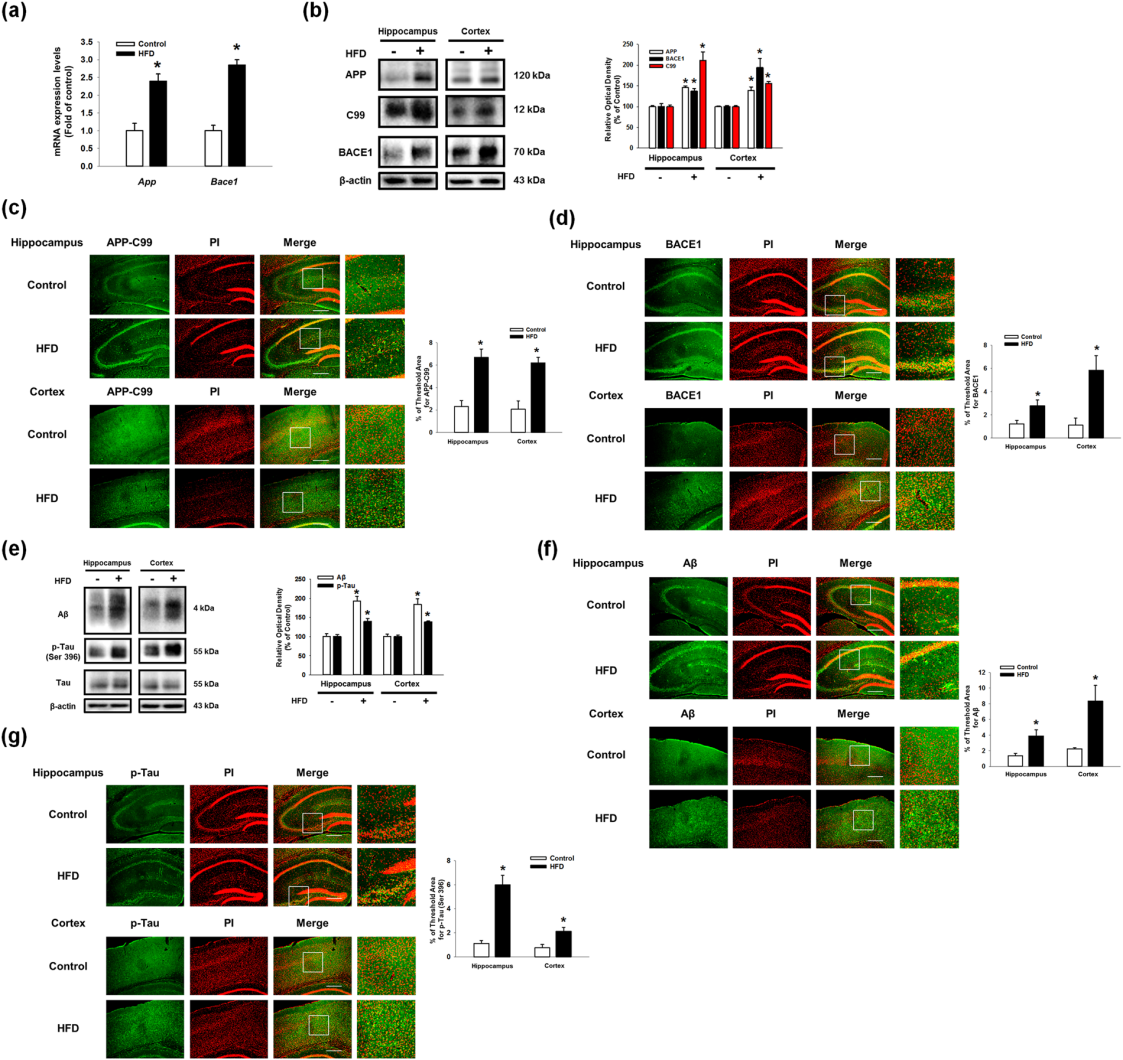
Effect of HFD on the regulation of Aβ regulating proteins expressions and Aβ production in the mouse brain. (**a**) Brains of 13 weeks old C57BL/6 mouse, which were fed with HFD for 8 weeks, were extracted. mRNA expressions of *APP* and *BACE1* in brain samples were analyzed by quantitative real time PCR. Data were normalized by *ACTB* mRNA expression level. Data are reported as a mean ± S.E.M. *n* = *4*. (**b**) Extracted brains were cryo-sectioned coronally, and separated into hippocampus and cortex. The expressions of APP, C99, BACE1 and β-actin in hippocampus and cortex regions were analyzed by western blot. Data are reported as a mean ± S.E.M. *n* = *5*. (**c**,**d**) Brain samples for immunohistochemistry were immunostained with C99 and BACE1 antibodies and PI. Images shown in result are representative. All scale bars, 150 μm (magnification, ×200). (**e**) Aβ, p-Tau (Ser396), Tau and β-actin protein expression levels in hippocampus and cortex regions of brain were assessed with western blot. (**f**,**g**) Brain samples for immunohistochemistry were immunostained with Aβ and p-Tau (Ser396) antibodies and PI. Image shown in result are representative. All scale bars, 150 μm (magnification, ×200). Each blot result shown is representative image. Quantitative blot data are presented as a mean ± S.E.M. *n* = *5*. *p < 0.05 versus control.

**Figure 2:**
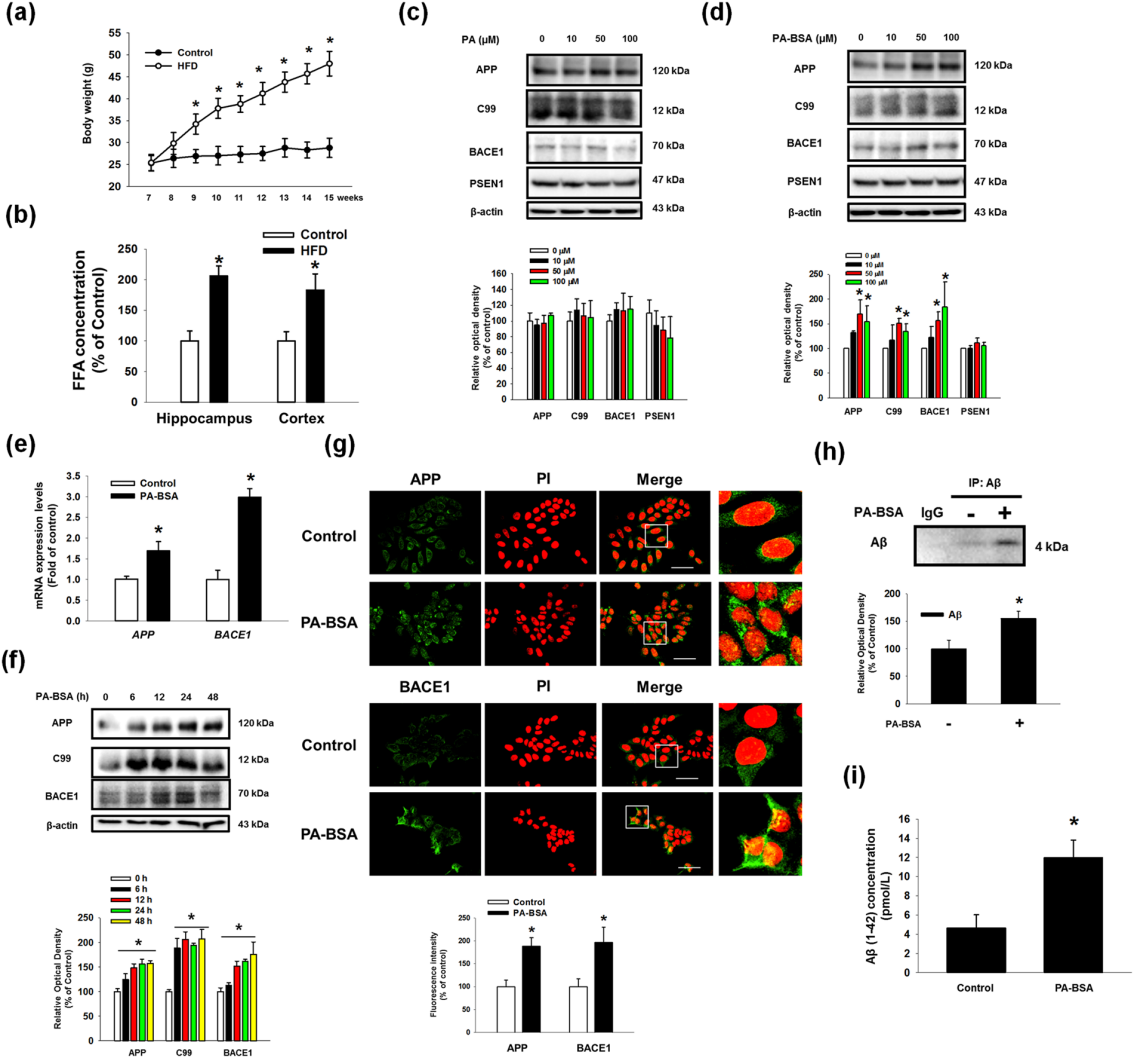
Effect of PA-BSA on the regulation of APP and BACE1 expressions and Aβ production in SK-N-MC cells. (**a**) The body weights of mice fed regular chow diet as a control or a HFD every week for 8 weeks. (**b**) Total FA concentration level in mouse brain samples was measured by using commercial kit described in Materials & Methods. Data are presented as a mean ± S.E.M. *n* = *5*. (**c**) SK-N-MC cells were incubated with various concentrations of PA (0–100 μM) for 24 h. APP, C99, BACE1, PSEN1 and β-actin proteins were assessed with western blot. *n* = *4*. (**d**) SK-N-MC cells were incubated with various concentrations of PA-BSA (0–100 μM) for 24 h. APP, C99, BACE1, PSEN1 and β-actin proteins were assessed with western blot. *n* = *4*. (**e**) SK-N-MCs were incubated with 50 μM of PA-BSA for 24 h. mRNA expressions of *APP* and *BACE1* were analyzed by quantitative real time PCR. Data were normalized by *GAPDH* mRNA expression level. Data are reported as a mean ± S.E.M. *n* = *4*. (**f**) SK-N-MC cells were treated with PA-BSA for 0–48 h. The expressions of APP, C99, BACE1, PSEN1 and β-actin were analyzed by western blot. *n* = *4*. (**g**) SK-N-MC cells were immunostained with APP and BACE1 antibodies and PI. Fluorescence images shown are representative. All scale bars, 50 μm (magnification, ×600). Fluorescence intensity data of APP and BACE1 is presented as a mean ± S.E.M. *n* = *5*. (**h**) Cells were incubated with 50 μM of PA-BSA for 72 h. Secreted Aβ in medium was analyzed by immunoprecipitation assay. Data are presented as a mean ± S.E.M. *n* = *3*. (**i**) Cells were incubated with 50 μM of PA-BSA for 48 h. Aβ concentration of medium samples were detected by using ELISA kit. Data are presented as a mean ± S.E.M. *n* = *6*. Each blot result shown is representative image. *p < 0.05 versus control.

To determine the effects of a high-fat diet (HFD) on Aβ production in the hippocampus and cortex, tissues from a mouse brain were analyzed by quantitative real time PCR, western blot and immunohistochemistry. First, we found that mRNA expression levels of *App* and *Bace1* in HFD fed mice were higher than those of regular chow-fed mice (Fig. 1a). As shown in Fig. 1b, APP and BACE1 expressions and the membrane bound C-terminal fragment C99 (C99) were increased in the hippocampus and cortex regions. Additionally, the number of C99 and BACE1-positive cells in the hippocampus and cortex regions in HFD brain tissues was greater than those of the control brain tissues (Fig. 1c and d). Aβ production and phosphorylation of Tau at the Ser396 residue were increased in the hippocampus and cortex of the HFD mice (Fig. 1e). In the immunohistochemistry results, a number of Aβ and phosphorylated Tau (Ser396)-positive cells were increased in the hippocampus and cortex regions in the brains of the HFD-fed mice (Fig. 1f and g). These results suggest that HFD stimulates the expressions of APP and BACE1 and Aβ production in mice brain. To confirm the effect of HFD on the biological parameter of the mice, we measured body weights of mice given a regular chow diet as a control or a HFD every week for 8 weeks. After 2 weeks of HFD feeding (9-week-old), the body weight of HFD-fed group mice was significantly higher than that of control group. In 8 weeks HFD feeding (15-week-old), the body weight of HFD-fed group were increased to 167% (Fig. 2a). In addition, we examined the concentration of total FA in both the control and brain samples of HFD-fed mice. As shown in the Fig. 2b, the concentrations of total FA in the hippocampus and cortex of brain samples of HFD-fed mice were increased to 206% and 183%, respectively. To determine the role of the genomic and non-genomic actions of PA in regulating the enzymes involved in Aβ production, we analyzed the protein expressions of APP, BACE1 and gamma secretase presenilin-1 (PSEN1) after SK-N-MC cells were treated with various concentrations of palmitic acid (PA) or bovine serum albumin-conjugated PA (PA-BSA). As shown in the Fig. 2c, free PA did not affect the expressions of APP, C99, BACE1 and PSEN1. However, the expressions of APP, BACE1 and C99 in the PA-BSA-treated cells were significantly higher than those in the control cells at 50 and 100 μM but not PSEN1 (Fig. 2d). We confirmed that PA-BSA treatment stimulated mRNA expressions of *APP* and *BACE1* in SK-N-MCs (Fig. 2e). In addition, PA-BSA increased APP and BACE1 expression as well as C99 in the SK-N-MC cells over 6 to 48 h (Fig. 2f). In the immunofluorescence staining results, the fluorescence intensities of APP and BACE1 in the PA-BSA-treated cells were increased to 188% and 197%, respectively (Fig. 2g). In addition, we performed immunoprecipitation with SK-N-MC-conditioned medium and Aβ antibody to confirm the regulation of Aβ production by PA-BSA. Our results show that treating SK-N-MC with PA-BSA increased Aβ production (Fig. 2h). To quantify the effect of PA-BSA on Aβ (1–42) production, we measured Aβ (1–42) concentrations in medium samples by using ELISA kit. Our data showed that the Aβ concentration levels in control medium and that of PA-BSA-treated medium are 4.6 and 12.0 pmol/L, respectively (Fig. 2i).

### PA-BSA stimulates the expressions of APP and BACE1 through the GPR40-mediated PI3K/Akt pathway

**Figure 3:**
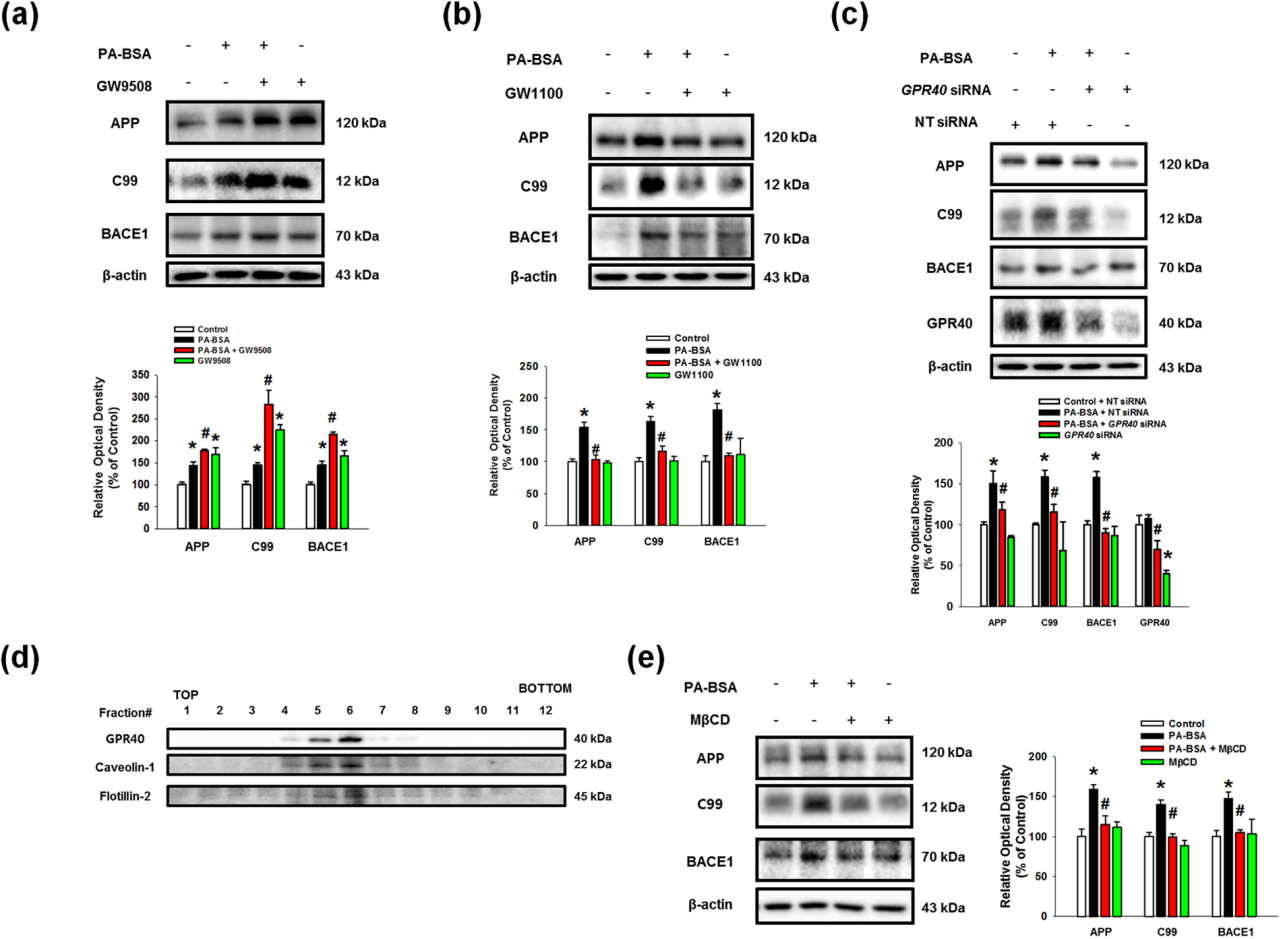
PA-BSA increased APP and BACE1 expressions through lipid raft-dependent GPR40 pathway. (**a**,**b**) SK-N-MC cells were pre-treated with GW9508 (10 μM) and GW1100 (10 μM) for 30 min prior to treatment of PA-BSA (50 μM) for 24 h. The expressions of APP, BACE1, C99 and β-actin were analyzed by western blot. Data are reported as a mean ± S.E.M. *n* = *4*. (**c**) *GPR40* and NT siRNAs were transfected to SK-N-MC cells for 12 h prior to incubation with PA-BSA (50 μM) for 24 h. The expressions of APP, C99, BACE1, GPR40 and β-actin were assessed with western blot. Data are reported as a mean ± S.E.M. *n* = *4*. (**d**) Sucrose gradient-fractionized samples were blotted with GPR40, Caveolin-1 and Flotillin-2 antibodies. (**e**) SK-N-MC cells were pre-treated with MβCD (500 μM) for 1 h prior to incubation with PA-BSA (50 μM) for 24 h. The expressions of APP, C99, BACE1 and β-actin were measured by western blot. Data are reported as a mean ± S.E.M. *n* = *4*. Each blot result shown is representative image. *p < *0.05* versus control, ^*#*^ *p* < 0.05 versus PA-BSA treatment.

**Figure 4:**
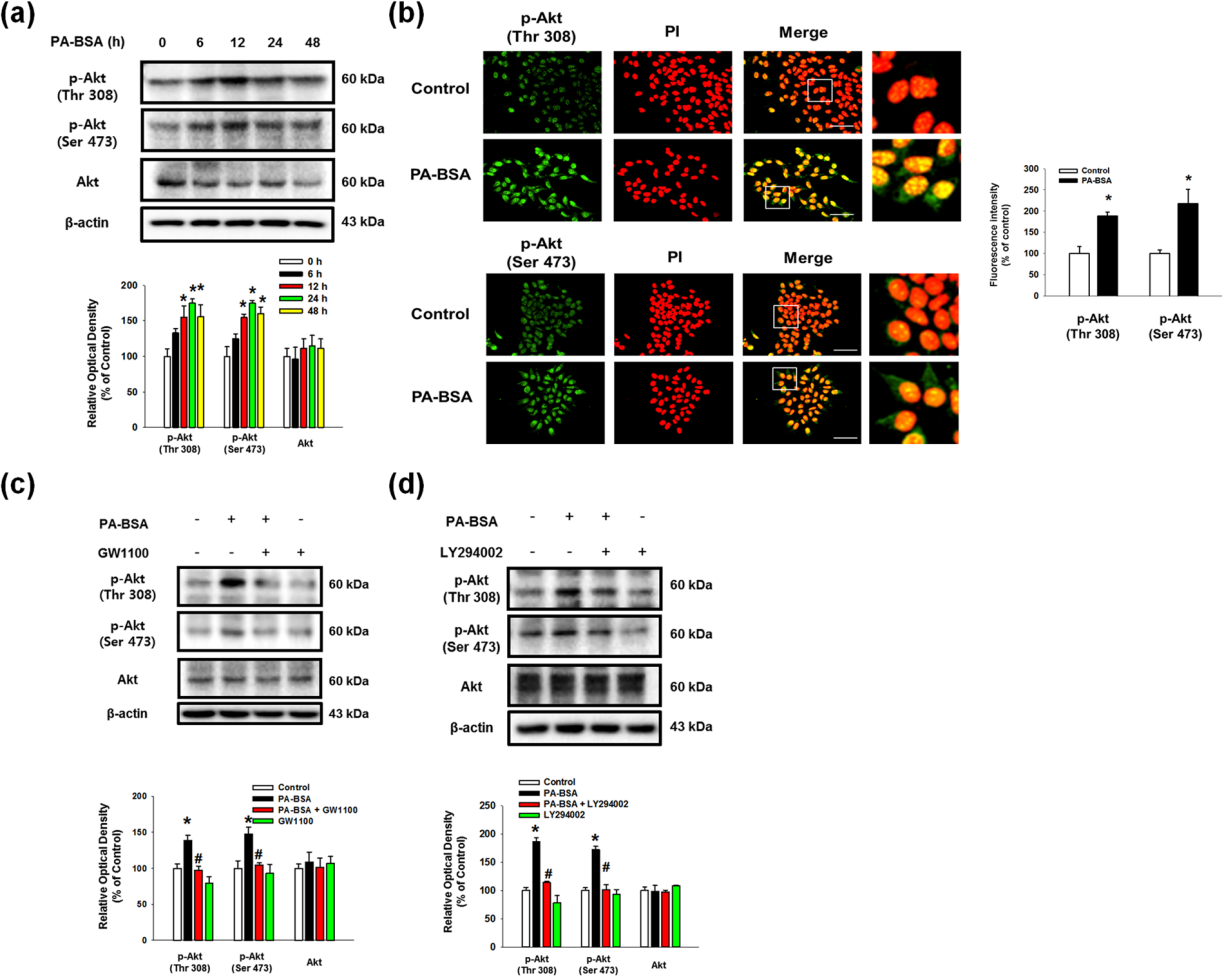
Role of PA-BSA in GPR40-mediated PI3K/Akt pathway. (**a**) SK-N-MC cells were exposed to PA-BSA (50 μM) for 0–48 h. p-Akt (Thr308 and Ser473), Akt and β-actin expressions were detected by western blot. Data are reported as a mean ± S.E.M. *n* = *4*. (**b**) SK-N-MC cells were incubated with PA-BSA (50 μM) for 24 h. Cells were immunostained with p-Akt (Thr308 and Ser473) antibodies and PI. All scale bars, 50 μm (magnification, ×600). Fluorescence intensity data of p-Akt (Thr308 and Ser473) was reported as a mean ± S.E.M. *n* = *5*. All fluorescence images are representative. (**c** and **d**) SK-N-MC cells were pre-treated with GW1100 (10 μM) and LY294002 (20 μM) for 30 min prior to incubation with PA-BSA (50 μM) for 24 h. Cells were blotted with p-Akt (Thr308 and Ser473), Akt and β-actin antibodies. Data are reported as a mean ± S.E.M. *n* = *4*. Each blot result shown is representative image. *p < *0.05* versus control, ^*#*^ *p* < 0.05 versus PA-BSA treatment.

To determine the role of the G-protein coupled receptors GPR40 and GPR120, also known free fatty acid (FFA) receptor 1 and 4, which are known to be activated by PA^27^, we investigated their mRNA expressions and receptor-induced signaling in PA-BSA treated SK-N-MC cells. We observed that the SK-N-MC cells contained *GPR40* and *GPR120* mRNAs (Sup. Fig. S1a), and the expressions of APP, BACE1 and C99 were augmented by pre-treatment with a GPR40/120 receptor agonist, GW9508, in the SK-N-MC cells (Fig. 3a). In addition, PA-BSA-induced APP and BACE1 expression and C99 were inhibited by the addition of the GPR40-specific inhibitor GW1100 (Fig. 3b) and by *GPR40* small interfering RNA (siRNA) transfection (Fig. 3c). However, the silencing of *GPR120* by siRNA transfection failed to regulate the APP and BACE1 expression and C99 (Sup. S1b). Figure 3d shows that the GPR40 receptor was expressed in the lipid raft fraction containing caveolin-1 and flotillin-2 (fractions #4–6). In addition, the disruption of the lipid raft by MβCD pre-treatment decreased the APP and BACE1 expression and C99 induced by PA-BSA (Fig. 3e). These results indicate that the expressions of APP and BACE1 induced by PA-BSA are dependent on the GPR40 receptor in the lipid raft. Next, we investigated the role of GPR40 signaling by PA-BSA in APP and BACE1 expression. As shown in Fig. 4a, the phosphorylation of Akt at Thr308 and Ser473 induced by PA-BSA was significantly higher than that of the control cells over 12 to 48 h of the PA-BSA treatment. Furthermore, the immunofluorescence staining results show that the fluorescence intensities of Akt at Thr308 and Ser473 residues in PA-BSA-treated cells were increased to 188% and 217%, respectively (Fig. 4b). In addition, we showed that the phosphorylation of Akt was inhibited by pre-treatment with GW1100 (Fig. 4c) and the phosphoinositide-3-kinase (PI3K) inhibitor LY294002, (Fig. 4d). Interestingly, our results show that the PA-BSA treatment did not affect intracellular calcium release, reactive oxygen species (ROS) production and PKC phosphorylation (Sup. Fig. S2a–S2c). In addition, GPR40 inhibition by GW1100 pretreatment suppressed p-GSK3β (Ser9) induced by PA-BSA (Sup. Fig. S3a). Overall, our findings indicate that PA-BSA activates GPR40-mediated PI3K/Akt signaling leading to the expressions of APP and BACE1.

### The Akt/mTOR/HIF-1α and Akt/NF-κB pathways are activated by the PA-BSA

**Figure 5:**
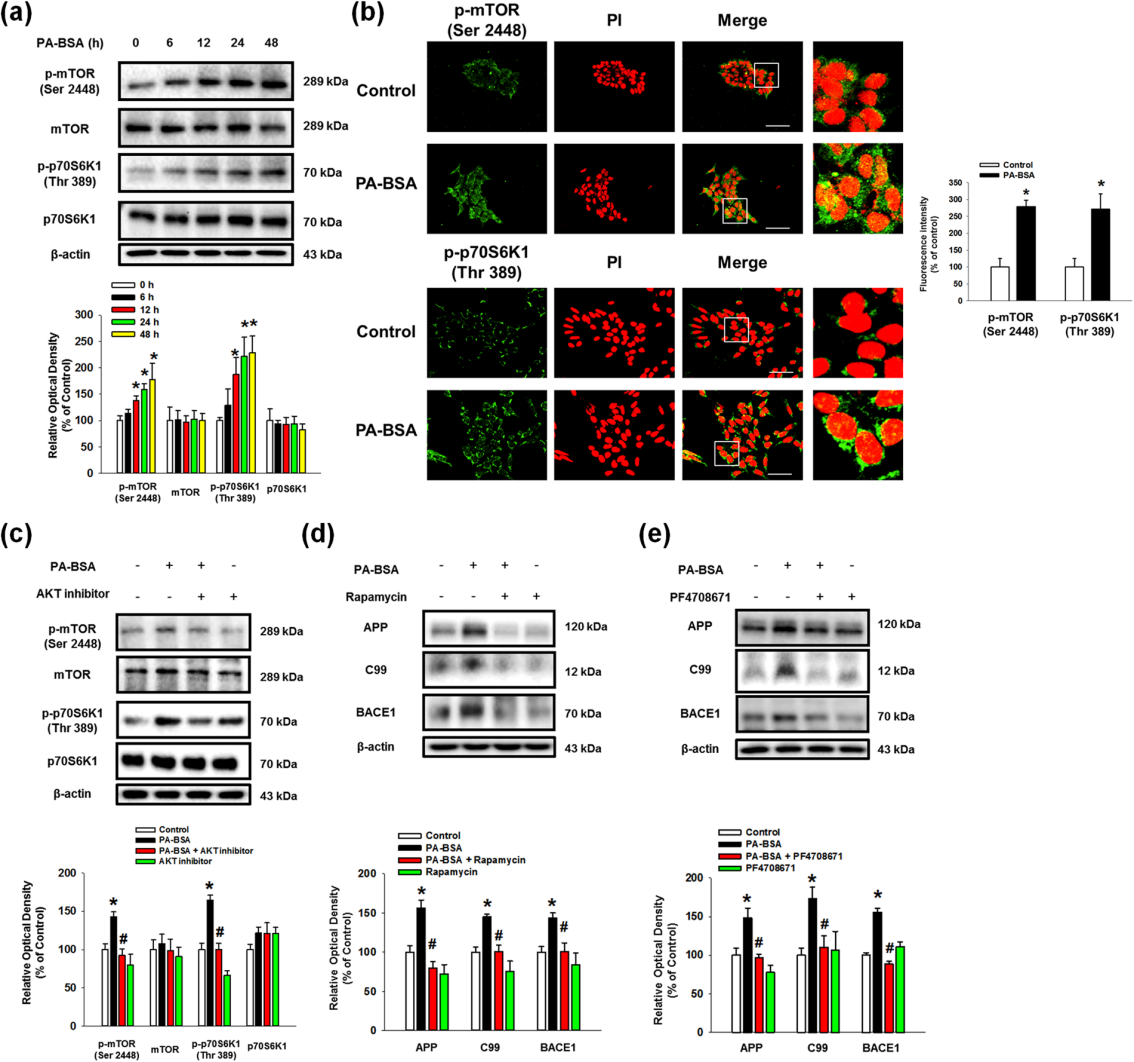
Role of mTOR activated by PA-BSA in APP and BACE1 expressions. (**a**) SK-N-MC cells were incubated with PA-BSA (50 μM) for 0–48 h. Cells were blotted with p-mTOR (Ser2448), mTOR, p-p70S6K1 (Thr389), p70S6K1 and β-actin antibodies. Data are presented as a mean ± S.E.M. *n* = *4*. (**b**) SK-N-MC cells were incubated with PA-BSA (50 μM) for 24 h. Cells were immunostained with p-mTOR (Ser2448) and p-p70S6K1 (Thr389) antibodies and PI. Each fluorescence image is representative. All scale bars, 50 μm (magnification, ×600). Fluorescence intensity data of p-mTOR (Ser2448) and p-p70S6K1 (Thr389) are reported as a mean ± S.E.M. *n* = *5*. (**c**) SK-N-MC cells were pre-treated with Akt inhibitor (1 μM) for 30 min prior to treatment of PA-BSA (50 μM) for 24 h. The expressions of p-mTOR (Ser2448), mTOR, p-p70S6K1 (Thr389), p70S6K1 and β-actin were analyzed by western blot. Data are reported as a mean ± S.E.M. *n* = *4*. (**d**,**e**) SK-N-MC cells were pre-treated with Rapamycin (10 nM) and PF4708671 (10 μM) for 30 min prior to incubation with PA-BSA (50 μM) for 24 h. The expressions of APP, C99, BACE1 and β-actin were analyzed by western blot. Data are presented as a mean ± S.E.M. *n* = *4*. Each blot image is representative. *p < *0.05* versus control, ^*#*^ *p* < 0.05 versus PA-BSA treatment.

**Figure 6:**
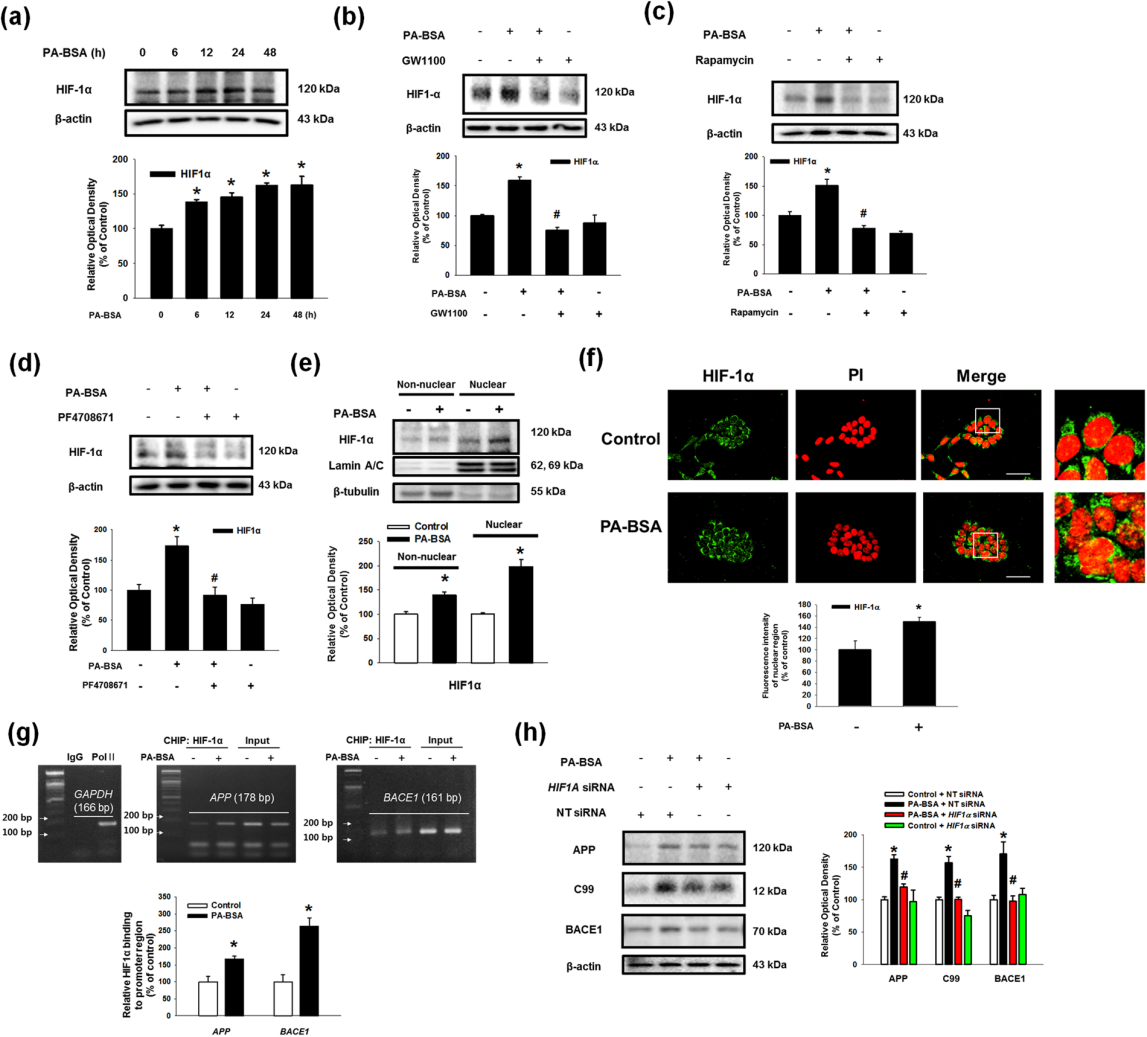
Role of mTOR/HIF-1α pathway activated by PA-BSA in APP and BACE1 expressions. (**a**) SK-N-MC cells were incubated with PA-BSA (50 μM) for 0–48 h. The expressions of HIF-1α and β-actin were detected by western blot. Data are presented as a mean ± S.E.M. *n* = *4*. (**b**–**d**) SK-N-MC cells were pre-treated with GW1100 (10 μM), Rapamycin (10 nM) and PF4708671 (10 μM) for 30 min prior to incubation with PA-BSA (50 μM). The expressions of HIF-1α and β-actin were analyzed by western blot. Data are reported as a mean ± S.E.M. *n* = *4*. (**e**) SK-N-MC cells were incubated with PA-BSA (50 μM) for 24 h. HIF-1α, Lamin A/C and β-tubulin in the non-nuclear and nuclear fractions were detected by western blot, respectively. The expressions of non-nuclear and nuclear protein were normalized by β-tubulin and Lamin A/C respectively. (**f**) SK-N-MC cells were immunostained with HIF-1α antibody and PI. Scale bars, 50 μm (magnification, ×600). Fluorescence intensity data of HIF-1α in the nuclear region was reported as a mean ± S.E.M. *n* = *5*. (**g**) DNA was immunoprecipitated with IgG, RNA polymerase II and HIF-1α antibodies. All samples including immunoprecipitation and input were amplified with the primers of *GAPDH*, *APP* and *BACE1* promoters. Quantitative data for HIF-1α binding to *APP* and *BACE1* promoters were analyzed by real time PCR. Data are presented as a mean ± S.E.M. *n* = *4*. (**h**) *HIF1A* and NT siRNAs were transfected to SK-N-MC cells for 12 h prior to incubation with PA-BSA for 24 h. The expressions of APP, C99, BACE1 and β-actin were detected by western blot. Data are reported as a mean ± S.E.M. *n* = *4*. Each blot image is representative. *p < *0.05* versus control, ^*#*^ *p* < 0.05 versus PA-BSA treatment.

**Figure 7:**
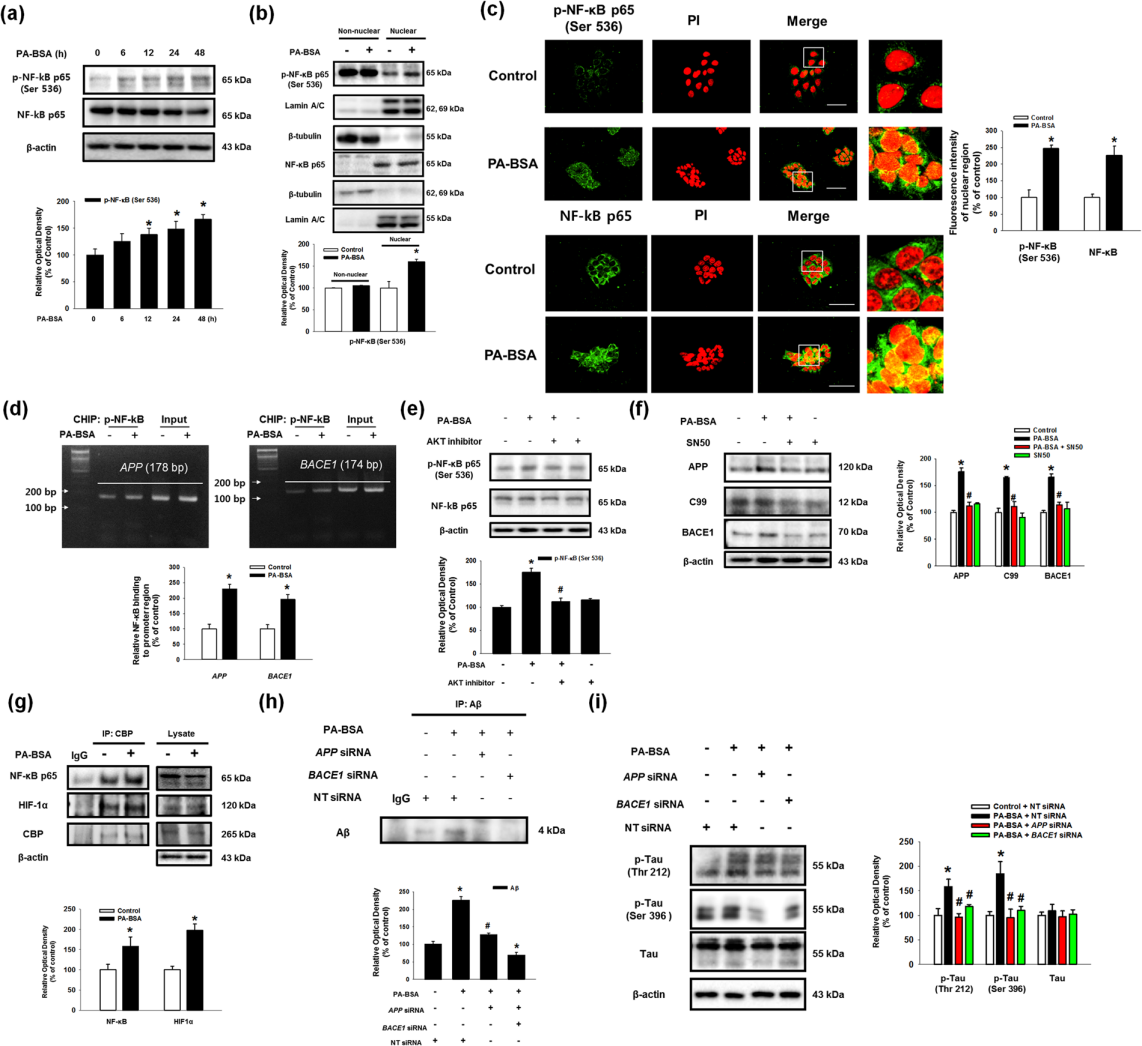
Role of Akt/NF-κB pathway induced by PA-BSA in APP and BACE1 expressions and Aβ production. (**a**) SK-N-MC cells were treated with PA-BSA (50 μM) for 0–48 h. The expressions of p-NF-κB p65 (Ser536), NF-κB p65 and β-actin were analyzed by western blot. Data are presented as a mean ± S.E.M. *n* = *4*. (**b**) SK-N-MC cells were treated with PA-BSA (50 μM) for 24 h. p-NF-Κb p65 (Ser536), NF-κB p65, Lamin A/C and β-tubulin in non-nuclear and nuclear fractions were detected by western blot. Data are reported as a mean ± S.E.M. *n* = *5*. (**c**) SK-N-MC cells were immunostained with p-NF-κB p65 (Ser536), NF-κB p65 antibodies and PI. Scale bars, 50 μm (magnification, ×600). Fluorescence intensity data of p-NF-κB p65 (Ser536) and NF-κB p65 in the nuclear region was reported as a mean ± S.E.M. *n* = *5*. (**d**) DNA was immunoprecipitated with IgG, RNA polymerase II and p-NF-κB p65 (Ser536) antibodies. All samples including immunoprecipitation and input were amplified with the primers of *GAPDH*, *APP* and *BACE1* promoters. Quantitative data for p-NF-κB p65 (Ser536) binding to *APP* and *BACE1* promoters were analyzed by real time PCR. Data are presented as a mean ± S.E.M. *n* = *4*. (**e**) SK-N-MC cells were pre-treated with Akt inhibitor (1 μM) for 30 min prior to PA-BSA (50 μM) for 24 h. Cells were blotted with p-NF-κB p65, NF-κB p65 and β-actin antibodies. Data are reported as a mean ± S.E.M. *n* = *4*. (**f**) SK-N-MC cells were pre-treated SN50 (5 μM) for 30 min prior to PA-BSA (50 μM) for 24 h. The expressions of APP, C99, BACE1 and β-actin were analyzed by western blot. Data are reported as a mean ± S.E.M. *n* = *4*. (**g**) Cell lysates were immunoprecipitated with CBP antibody. NF-κB p65, HIF-1α, CBP and β-actin in samples for immunoprecipitation and input were analyzed by western blot. Data are reported as a mean ± S.E.M. *n* = *3*. (**h**) SK-N-MC cells were incubated with PA-BSA (50 μM) for 72 h. Secreted Aβ in medium was assessed with immunoprecipitation assay. Data are presented as a mean ± S.E.M. *n* = *3*. (**i**) Cells were transfected with APP and BACE1 siRNAs for 12 h prior to PA-BSA treatment for 24 h. Cells were blotted with p-Tau (Thr212), p-Tau (Ser396), Tau and β-actin. Data are presented as a mean ± S.E.M. *n* = *3*. Each blot result shown is representative image. *p < 0.05 versus control, ^*#*^ *p* < 0.05 versus PA-BSA treatment.

Subsequently, we found that phosphorylation of the mammalian target of rapamycin (mTOR) and p70 S6 kinase 1 (p70S6K1) was increased by PA-BSA in a time-dependent manner (Fig. 5a). Immunofluorescence results show that the fluorescence intensities of p-mTOR (Ser2448) and p-p70S6K1 (Thr389) in PA-BSA treated cells were increased to 279% and 271%, respectively (Fig. 5b). Our results show that the phosphorylation of p70S6K1 induced by PA-BSA was inhibited by pre-treatment with GW1100 (Sup. Fig. S3b). As shown in the Fig. 5c, increased mTOR and p70S6K1 phosphorylation by PA-BSA was blocked by pre-treatment with the Akt inhibitor. In addition, the PA-BSA-induced APP and BACE1 expression and C99 were abolished by pre-treatments with the mTOR inhibitor rapamycin and the p70S6K1 inhibitor PF4708671 (Figs. 5d and 5e). These findings imply that PA-BSA activates mTOR complex 1 (mTORC1)-p70S6K1 signaling through Akt activation. Furthermore, we found that the maximum increase in hypoxia-inducible factor 1-alpha (HIF-1α) expression was observed after 24 h of PA-BSA treatment (Fig. 6a), and PA-BSA-stimulated HIF-1α expression was abolished by pre-treatment with GW1100, rapamycin and PF4708671 (Fig. 6b–d). Our results further revealed that the expression of HIF-1α in the non-nuclear and nuclear fractions was increased by PA-BSA (Fig. 6e), and the fluorescence intensity of HIF-1α in the PI region was increased to 149% by PA-BSA (Fig. 6f). The CHIP assay results show that PA-BSA significantly enhanced the binding of HIF-1α to the promoter regions of the *APP* and *BACE1* genes (Fig. 6g). The silencing of HIF-1α by *HIF1A* siRNA transfection inhibited the APP and BACE1 expressions and C99 induced by PA-BSA (Fig. 6h). The transfections of *HIF1A* siRNA decreased the HIF-1α expression induced by PA-BSA (Sup. Fig. S4). Furthermore, we investigated the role of nuclear factor kappa-light-chain-enhancer of activated B cells (NF-κB), in the regulation of APP and BACE1 expression. Our results show that the phosphorylation of NF-κB in SK-N-MC cells treated with PA-BSA was increased in a time-dependent manner over 6 to 48 h (Fig. 7a). In addition, we showed that the expressions of phosphorylated NF-κB at Ser536 and NF-κB in the nuclear fraction were increased by PA-BSA (Fig. 7b). We further determined that the fluorescence intensities of p-NF-κB and NF-κB in the PA-BSA treated cells increased to 247% and 226% of the control cells, respectively (Fig. 7c). The CHIP assay results show that PA-BSA stimulated the binding of NF-κB to the *APP* and *BACE1* promoters (Fig. 7d). As shown in the Supplementary Fig. S3c and Fig. 7e, phosphorylation of NF-κB by PA-BSA was reduced by the addition of GW1100 or the Akt inhibitor. In addition, Akt inhibitor pretreatment suppressed GSK3β phosphorylation at Ser9 residue as well as Tau phosphorylations at Thr212 and Ser396 residues (Sup. Fig. S5). The APP and BACE1 expressions and C99 stimulated by PA-BSA were inhibited by the addition of the NF-κB inhibitor SN50 (Fig. 7f). Moreover, the immunoprecipitation results show that HIF-1α and NF-κB interacted with a transcriptional coactivator CREB-binding protein (CBP) in the SK-N-MC cells, and the formation of the HIF-1α/CBP/NF-κB complex was enhanced by PA-BSA (Fig. 7g). As shown in the Figs. 7h and 7i, Aβ secretion and Tau phosphorylations at Thr212 and Ser396 residues induced by PA-BSA were reduced by *APP* and *BACE1* siRNAs transfections in SK-N-MC cells. The transfections of *APP* and *BACE1*-siRNAs decreased the APP and BACE1 expression induced by PA-BSA (Sup. Figs. S6a and S6b). Taken together, these results indicate that HIF-1α and NF-κB induced by PA-BSA stimulate the expressions of APP and BACE1 in a cooperative manner leading to Aβ production in SK-N-MC cells.

## Discussion

**Figure 8:**
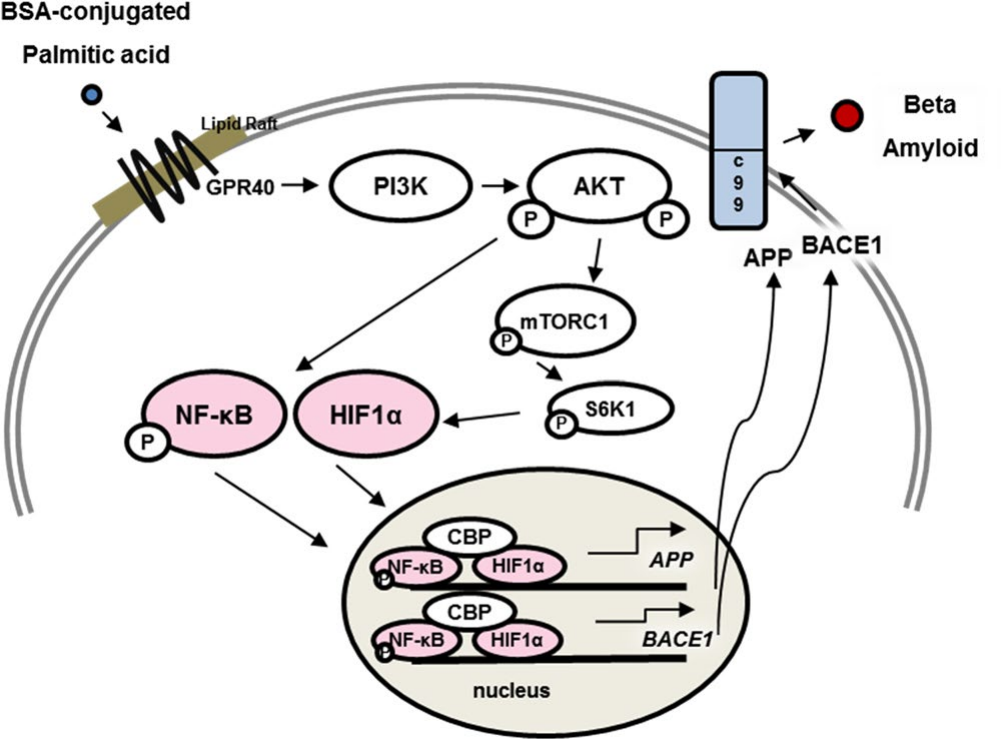
The schematic model for mechanism of non-genomic actions of PA-BSA in SK-N-MC cells. PA-BSA increases APP and BACE1 expressions via GPR40-mediated Akt-/NF-κB and Akt/mTORC1/HIF-1α pathways, followed by Aβ production.

In the present study, we showed that PA-BSA stimulates the expressions of APP and BACE1 through the Akt/mTOR/HIF-1α and Akt/NF-κB pathways in SK-N-MC cells (Fig. 8). Recent studies have shown that APP processing has an important role in HFD-induced AD-like changes in AD transgenic mice^10, 28^. Tucsek *et al*. reported that Aβ production was increased by HFD in normal C57BL/6 obese mice^29^. Consistent with that result, our results with a normal C57BL/6 mouse model showed that HFD stimulated the expressions of APP as well as the APP processing enzyme BACE1 in the mouse brain. These findings indicate the possibility that HFD stimulates APP expression as well as APP processing. Our data showed that HFD feeding increases FA accumulation in the brain as well as the body weight. A previous investigation also demonstrated that the HFD increases the total body weight^30^. Those findings indicate that there are close relationships between the increase of body weight and FA accumulation induced by HFD feeding in the mice brain. Furthermore, several studies have shown the effects of lipid metabolites on AD pathogenesis which suggests that elevated FAs and oxysterol levels stimulate Aβ and Tau polymerization in various types of cells including neuronal cells^31–33^. In rodents, a diet high in saturated FAs was found to be more detrimental than a high-cholesterol diet^11^. Other rodent studies have shown that trans- and saturated-FAs lead to a particularly robust increase in Aβ^22^. Conversely, diets with high levels of omega 3 polyunsaturated fatty acids are associated with decreased Aβ levels, and one study found that a diet low in fat and high in oleic acid reduced the Aβ levels and pathology in transgenic mice^34, 35^.

Among all the FAs, palmitic acid (PA) is an abundant saturated FA existing in the human body and closely linked to metabolic diseases and AD. A key finding of our study is that PA conjugated with bovine serum albumin (PA-BSA) has a significant ability to directly increase *APP* and *BACE1* but not *PSEN1* mRNA expressions and to promote amyloidogenesis in SK-N-MC cells. This result differs from that in a previous report which revealed that the genomic actions mediated by free PA actually do not evoke BACE1 expression, APP processing, and Tau hyperphosphorylation in primary neurons^18, 36, 37^. Instead, the investigators reported that conditioned medium from astrocytes, which have a higher capacity to metabolize free PA, induces BACE1 upregulation and C99 accumulation in primary neurons^18^. Although the reasons for this discrepancy regarding the functional role of PA in the regulation of AD remains unknown, one possibility in the present study is that the intra-cellular signaling by free PA is different from the extra-cellular signaling by PA-BSA. Thus, our results indicate that the pathophysiological actions of extra-cellular PAs on AD occurrence could be mediated by non-genomic actions which show that extra-cellular signaling triggered by PA-BSA may directly contribute to APP and BACE1 expression and to amyloidogenesis. There are few studies investigating whether PA is in a free or albumin-conjugated form in the brain. However, it has been reported that more than 99% of FFAs exist in albumin-conjugated form in blood plasma, and FFA is permeable to blood brain barrier^38–40^. In addition, several investigators reported that HFD feeding stimulates FA uptake into the brain from blood plasma^41, 42^. Therefore, we suggest a possibility that most of PA in brain may be in an albumin-conjugated form and HFD feeding increases absorbance of PA into the brain from plasma. To the best of our knowledge, this is the first study to provide evidence on whether PA regulating AD pathogenesis is in the free form or in the albumin-conjugated form.

To know how extra-cellular PA induces the expressions of APP and BACE1, we further investigated the role of PA-BSA in the activation of two free-fatty-acid receptors, GPR40 and GPR120^27^. Our results show that APP and BACE1 expressions induced by PA-BSA was dependent on GPR40 but not on GPR120 which is consistent with previous reports. A previous study showed that GPR40 was expressed in various regions of the brain including the cerebellum, hippocampus, and cerebral cortex; however, GPR120 was not found in any brain regions^43^. Moreover, it is well documented that GPR40 has a critical role in neurogenesis and neuronal development as well as in sporadic AD occurrence^44^. There are many reports suggesting that the activation of GPR40 induces various signaling pathways including the Ca^2+^, ROS and PI3K/Akt-dependent signaling pathways; however, the role of specific signaling in the regulation of cell functions appears to be depend on the cell type and specific ligand^22, 45–49^. In the nervous system, docosahexaenoic acid and linoleic acid promote neuronal differentiation and sensory neuron activation through GPR40-mediated calcium signaling^45, 48^. Previous research showed sodium PA dephosphorylated GSK3β at Ser9 residue^50^. Although a GSK-3β has been recognized as a kinase involved in AD pathogenesis, our data showed that PA-BSA phosphorylated and inhibited GSK3β via GPR40/Akt pathway. It has been well documented that Akt phosphorylates GSK3β at Ser9 residue and inactivates GSK3β^51^. Those findings indicate that PA-BSA induces Akt-dependent GSK3β phosphorylation and inactivation, which is associated with non-genomic action of PA-BSA. Moreover, we confirmed that GPR40 exists in the lipid raft. Because APP processing is related to membrane rafts^52^, we showed that lipid raft disruption incrementally repressed PA-BSA-induced APP, C99 and BACE1. Consistent with these findings, our data indicate that the induction of APP and BACE1 expressions by PA-BSA is dependent on PI3K/Akt signaling. Furthermore, we did not find any difference in intra-cellular calcium, PKC phosphorylation and ROS levels after the PA-BSA treatment. In contrast, previous results have shown compelling evidence that free PA activates intra-cellular calcium and STAT3 pathways in astrocytes to enhance the amyloidogenic processing of APP^18^. Thus, the results in this study indicate that extra-cellular PA uniquely regulates the PI3K/Akt signaling pathway to induce amyloidogenesis which differs from the genomic activation of free PA.

In addition, several studies have shown that the activation of PI3K/Akt signaling in the nervous system is important for AD pathogenesis through Tau phosphorylation^53, 54^. One study investigated the role of the PI3K/Akt pathway in APP processing suggesting that PI3K/Akt activation is associated with BACE1 expression in the brains of HFD-fed mice^55^. This finding is consistent with our data showing that the PI3K/Akt pathway is a key regulator of APP and BACE1 expressions in SK-N-MC cells treated with PA-BSA. In addition, we presented that phosphorylation of Tau induced by PA-BSA were controlled by activation of Akt and up-regulation of APP and BACE1. It has been a previous report showing the direct phosphorylation of Tau by Akt activation^53^. But, our investigation suggests the possibility that Tau phosphorylation induced by PA-BSA is mainly regulated by Akt-induced APP and BACE1 expression rather than direct phosphorylation of Akt. One of the responsive molecules associated with PI3K/Akt is the mTOR, which is hyper-activated in both mild and severe AD patients^56^. Our data show that the Akt/mTOR/p70S6K1 pathway induced by PA-BSA coupling with GPR40 directly stimulates APP and BACE1 expressions. Although a detailed mechanism of mTOR promoting AD pathogenesis remains a topic of much debate, the mTOR inhibitor has been well studied for its action to ameliorate symptoms in AD models^56^. In fact, it was previously shown that mTOR induces AD pathogenesis through auto-phagosomal accumulation^57, 58^. Translational regulation is one of the main functions of mTOR; however, the role of mTOR in the direct regulation of APP and BACE1 expressions has not been accurately elucidated. One report showed that BACE1 expression in primary neurons is mainly controlled by mTOR and p70S6K^59^. In addition, a study by *Caccamo et al*. showed the relevance of p70S6K1 and BACE1 in AD neuropathology^60^. Therefore, these earlier and present findings suggest that the mTOR/p70S6K1-mediated translational regulation of APP and BACE1 has an important role in Aβ production.

Furthermore, we showed that extra-cellular PA acting on mTOR induces the transcriptional occupancy of HIF-1α in the APP and BACE1 promoters. Although there are many reports presenting the significance of HIF-1α in AD pathogenesis, the role of HIF-1α in AD progression remains controversial. For instance, some researchers have proposed that hypoxic preconditioning could prevent the deterioration of neuronal cells in AD as an alternative therapy^61, 62^. However, many contradictory results have also been reported showing that the manipulation of hypoxic pathways have many different outcomes^62^. For instance, we have previously shown that HIF-1α binding to the *BACE1* promoter induces BACE1 expression and results in increased Aβ production in neuroblastoma cells^63^. Moreover, hypoxia-mediated HIF-1α signaling is involved in the amyloidogenic processing of the amyloid precursor protein, and subsequent downstream events influence the activation of the pro-death gene BNIP3, thus leading to an increased incidence of AD and neurodegeneration after cerebral ischemia and stroke^62, 64^. In agreement with our current findings, HIF-1α activation induced by FAs and HFD has been shown to act as a central mediator of angiogenesis and metabolic process^65, 66^. These results are consistent with the notion that patients who suffer from ischemia are more sensitive to AD development^67, 68^. To the best of our knowledge, this is the first study to provide evidence that extra-cellular PA acting on GPR40 triggers HIF-1α binding to the *APP* and *BACE1* promoters via mTOR activation.

On the other hand, we also showed that the phosphorylation of another key transcription factor, NF-κB, stimulated APP and BACE1 expressions in SK-N-MC cells treated with PA-BSA. Despite the fact that post-mitotic CNS neurons have been reported to show NF-κB responses that are exceedingly attenuated^69^, evidence has suggested that NF-κB is activated in neurons by non-immunological stimuli including developmental signals or stress in the adult brain^70^. In addition, previous work has shown that activated NF-κB is responsible for AD pathogenesis leading to an increase in BACE1 activity as well as Aβ production^71^. Moreover, the phosphorylation of NF-κB also influences the DNA binding affinity and transcriptional efficacy of NF-κB^72^. Consistently, earlier work also has shown that activated NF-κB directly binds to the promoters of APP and BACE1 stimulating their expression levels^73–75^. Therefore, we suggest that HIF-1α and NF-κB activated by Akt cooperatively promote APP and BACE1 expression in SK-N-MC cells treated with PA-BSA. Our result is consistent with the notion that continual active Akt overexpression stimulates the transactivation of HIF-1α and NF-κB^76^, although there are unresolved issues about the crosstalk between HIF-1α and NF-κB. Importantly, we further confirmed that HIF-1α and NF-κB activated by PA-BSA form a complex together through CBP. Collectively, these results provide important evidence of a cellular mechanism for the extra-cellular PA signaling pathway by which the expressions of APP and BACE1 are enhanced in a cooperative manner through HIF-1α and NF-κB activation resulting in Aβ production.

Taken together, present study indicates high concentration level of PA-BSA increases the potential of AD occurrence although not all obese patients have AD pathology including Aβ accumulation and Tau hyper-phosphorylation. Many researchers also demonstrated that AD pathogenesis is controlled by Aβ clearance and Tau phosphorylation by kinases as well as Aβ production^77, 78^. Hence, we suggest that those risk factors can affect the AD occurrence in obese patient with high PA-BSA level. Moreover, our observations provide insight into the essential role of extra-cellular PA signaling as a physiological regulator in Aβ production. This study offers a meaningful approach by which to investigate the relationship between obesity and AD. Our results can also lead to further research connecting metabolic and neurodegenerative diseases. In conclusion, this study showed that PA-BSA stimulates APP and BACE1 expressions as well as Aβ generation through the GPR40-mediated Akt/mTOR/HIF-α and Akt/NF-κB pathways in SK-N-MC cells.

## Materials and Methods

### Materials

The human neuroblastoma cell line SK-N-MC was obtained from Korean Cell Line Bank (Seoul, Korea). The antibodies of p-Akt (Thr308), p-Akt (Ser473), Akt, mTOR, Caveolin-1, Flotillin-2, p-Tau (Ser396), Tau﻿, p-NF-κB p65 (Ser536), NF-κB p65, Lamin A/C, CBP and β-actin were purchased from Santa Cruz Biotechnology (Santa Cruz, CA, USA). The antibodies of p-mTOR (Ser2448), p-p70S6K1 (Thr389) and p70S6K1, were acquired from Cell Signaling Technology (Beverly, MA, USA). Aβ, BACE1, HIF-1α and GPR40 antibodies were obtained from Abcam (Cambridge, MA, USA). The C99 antibody was purchased from EMD Millipore (Darmstadt, Germany). Horse radish peroxidase (HRP)-conjugated IgG was obtained from Jackson Immunoresearch (West Groove, PA, USA). PA, BSA, GW9508, ionomycin, PF4708671, LY294002 and rapamycin were purchased from Sigma Chemical Company (St. Louis, MO, USA). The Akt inhibitor, GW1100 and SN50 used here were purchased from Calbiochem (La Jolla, CA, USA). siRNAs for *GPR40*, *GPR120*, *APP*, *BACE1, HIF-1α* and non-targeting were obtained from Dharmacon (Lafayette, CO, USA).

### Culture of SK-N-MC cells

SK-N-MC cells were cultured without a feeder layer in high-glucose Dulbecco’s Modified Eagle Medium (DMEM; Life Technologies, Gaithersburg, MD, USA) supplemented with 1% penicillin and streptomycin (Life Technologies), and 10% FBS (Hyclone, Logan, UT, USA). Cells were grown in 60π culture dishes in an incubator maintained at 37 °C with 5% CO_2_. The medium was replaced with DMEM supplemented with 1% penicillin and streptomycin and 2% of serum replacement (SR; Life Technolog) 24 h before the experiments. Cells were maintained in 2% SR in DMEM containing 1% antibiotics and proper agents for the indicated time points.

### Preparation of brain tissue of the HFD-induced obese mouse model

To induce obesity through a diet, five-week-old male C57BL/6 mice were fed either a regular chow diet or a HFD (20% carbohydrate, 60% fat, 20% protein; D12492; Research Diets Inc., New Brunswick, NJ, USA) for 8 weeks. Mice were maintained under a 12 h light-dark cycle and had free access to water in a specific pathogen-free barrier facility. Mice were randomly assigned. The experiments were performed according to the “Guide for Animal Experiments” (Edited by the Korean Academy of Medical Sciences) and approved by the Institutional Animal Care and Use Committee of Seoul National University (SNU121119–3). The brains were removed and post-fixed by 4% paraformaldehyde in 0.1 M PBS for 12 h. The brain tissues were cryo-protected by overnight infusion with 90% sucrose, and 30 µm-thick coronal sections were serially cut using a cryostat (Leica, Deerfield, Germany). The sections were transferred to six-well plates containing PBS for further processing.

### Preparation of BSA-conjugated PA

500 mM of PA was melted in 1 ml of ethanol at 70 °C. 10% FFA-free BSA was dissolved in DMEM at 37 °C and subsequently filtered through a Minisart syringe filter (Sartorius Stedim, Heidelberg, Germany). 10 μl of PA was inserted in 1 ml of BSA while being vortexed. PA-BSA was incubated in a water bath at 55 °C for 15 min and conjugated in a water sonicator for 15 min. Consecutively, PA-BSA was incubated in a water bath at 55 °C for 15 min again. The vehicle control was prepared by mixing 10 μl of ethanol with 1 ml of 10% FFA-free BSA media. PA-BSA and the vehicle were stored at −20 °C and melted in a water bath at 55 °C for 15 min for further treatment. The molar ratio of PA-BSA is 3.3:1 (PA: BSA).

### Western blot analysis

Cells were washed once with cold PBS, detached from the culture dishes with a scraper and gathered by centrifugation (15,000 rpm, 4 °C, 5 min). Harvested cells and brain tissues were lysed by RIPA lysis buffer (20 mM HEPES (pH 7.5), 1% IGEPAL, 0.1% SDS, 0.5% deoxycholic acid, 150 mM sodium chloride) with 1% of protease/phosphatase inhibitor (Life technologies) and incubated for 30 min on ice. The lysates were then cleared by centrifugation (15,000 rpm, 4 °C, 30 min). The protein concentration was measured with a protein assay using bicinchoninic acid (BCA). Samples containing 10 ug of protein were prepared for 8 ~ 15% sodium dodecyl sulfate polyacrylamide gel electrophoresis (SDS-PAGE) and then transferred to a polyvinylidene fluoride (PVDF) membrane. These membranes were washed with TBST (10 mM Tris-HCl (pH 7.6), 150 mM NaCl, and 0.1% Tween-20) and blocked with 5% skim milk (Life technologies) dissolved in TBST for 30 min. The blocked membranes were washed with TBST three times every 10 min and incubated with primary antibody overnight at 4 °C. The membranes were subsequently washed and incubated with HRP-conjugated secondary antibody at 4 °C for 4 h. The western blotting bands were visualized by means of chemiluminescence (Bio-Rad, Hercules, CA, USA). Statistical analysis was carried out using the ImageJ software (developed by Wayne Rasband, National Institutes of Health, Bethesda, MD, USA; http://rsb.info.nih.go.kr/ij/). Full-length gels images of blot data are presented in the Supplementary information (Sup. Figs. S7–S14).

### Reverse transcription (RT)-polymerase chain reaction and real-time PCR/PCR

RNA was extracted from the cells using a MiniBEST Universal RNA Extraction Kit (TaKaRa, Otsu, Shinga, Japan). Reverse transcription was carried out using 1 μg of RNA with a Maxime RT-PCR PreMix kit (Intron Biotechnology, Seongnam, Korea) with the oligo(dT18) primers. RT was performed in 45 °C for 60 min for cDNA synthesis and at 95 °C for 5 min for the RNase inactivation step. 2 μl of the RT products was then amplified using QuantiSpeed SYBR kits (Life technologies, NY, USA). Real-time quantification of RNA targets was performed with a Rotor-Gene 6000 real-time thermal cycling system (Corbett Research, NSW, Australia). The reaction mixture (20 μl) contained 200 ng of RT product, 0.05 mM of each primer, and appropriate amounts of enzymes and fluorescent dyes, as recommended by the supplier. PCR and real-time PCR were performed as follows: 15 min at 95 °C for DNA polymerase activation; 15 sec at 95 °C for denaturing; and 40 cycles of 15 sec at 94 °C, 30 sec at 54 °C, and then 30 sec at 72 °C. Data was collected during an extension step (30 sec at 72 °C) and the analysis was conducted using the Rotor-Gene 6000 Series Software package version 1.7 and Sigma Plot version 10.0. Following the real-time PCR step, a melting curve analysis was conducted to verify the specificity and identity of the PCR products. Normalization was performed using β-actin as an endogenous control. Sequences of the primers used are described in Supplementary Table S1.

### Measurement of calcium influx

Changes in Ca^2+^ were observed using Fluo 3-AM dissolved in DMSO. The cells, seeded in confocal dishes, were washed once with PBS, incubated in DMEM containing 3 μM of Fluo 3-AM with 5% CO_2_ at 37 °C for 1 h, washed once with PBS and scanned every second using confocal microscopy (×400) (FluoView^TM^ 300, Olympus, Tokyo, Japan). The fluorescence was excited at 488 nm and the emitted light was read at 515 nm. In order to verify the assay, cells were treated with ionomycin as a positive control. Analyses of Ca^2+^ were conducted with FluoView^TM^ software. Ca^2+^ levels are expressed as the relative fluorescence intensity (RFI).

### Measurements of released ROS levels

CM-H_2_DCFDA (DCF-DA) was used to detect the intracellular H_2_O_2_. The cells were plated on six-well dishes and were washed with PBS and incubated in the dark with DMEM containing DCF-DA (10 μM) for 1 h at 37 °C with 5% CO_2_. 100 μl of the cell suspension was loaded into a 96-well plate and assessed using a luminometer (Victor3, Perkin-Elmer, MA, USA) at an excitation and emission wavelength of 485 and 535 nm, respectively.

### siRNA Transfection

Cells were grown until 70% confluence and then transfected for 24 h with *APP*, *BACE1*, *GPR40*, *GPR120* and non-targeting siRNAs (Dharmacon, Lafayette, CO, USA) using TurboFect^TM^ transfection reagent (Thermo Fisher, Rockford, IL, USA) in 2% SR in DMEM. After 24 h of incubation, the culture media were replaced with transfection mixture-free and 2% SR in DMEM and the cells were maintained for 24 h. The siRNAs sequences used are described in Supplementary Table S2.

### Nuclear Fractionation

Prior to harvesting the cells, they were washed once with cold PBS. The harvested cells were suspended in nuclear fraction buffer (137 mM NaCl, 8.1 mM Na_2_HPO_4_, 2.7 mM KCl, 1.5 mM KH_2_PO_4_, 2.5 mM EDTA, 1 mM dithiothreitol, 0.1 mM PMSF, and 10 mg/ml leupeptin [pH 7.5]). Suspended cells were lysed mechanically through homogenization with a 23-gauge needle. Cell lysates were centrifuged at 8,000 rpm for 5 min at 4 °C. The lysate supernatant as a non-nuclear fraction was collected. The obtained pellet, as a nuclear fraction, was then lysed with RIPA lysis buffer.

### Chromatin Immunoprecipitation (CHIP)

CHIP was performed with an EZ-ChIP-chromatin immunoprecipitation Kit (EMD Millipore) according to the manufacturer’s instructions. Chromatin-protein complexes were immunoprecipitated using the HIF-1α and NF-κB p65 antibodies. The normal IgG was used as a negative control. After overnight incubation, immune complexes were eluted with 200 μl (two times at 100 μl each) of an elution buffer (1% SDS, 50 mM Tris-HCl, pH 7.5, and 10 mM EDTA) and were then incubated with RNase for 1 h and 4 h with proteinase K at 65 °C. DNA was extracted and amplified by PCR using the *APP* and *BACE1* primers. As inputs, we used products that corresponded to PCR reactions containing 1% of the total chromatin extract used in the immunoprecipitation reactions. Sequences of primers for CHIP assay are described in the Supplementary Table S3.

### Determination of Aβ concentration

The Aβ (1–42) concentration level in medium sample was measured by commercial enzyme-linked immunosorbent assay (ELISA) kits (Wako Pure Chemical, Tokyo, Japan). SK-N-MCs were incubated with vehicle control or PA-BSA for 48 h. Medium samples were collected and centrifugated at 15,000 rpm for 5 min to remove the cell and debris. Supernatant samples were collected and prepared as ELISA samples. Aβ ELISA assay was performed according to manufacturer’s indication.

### Total Fatty Acid Quantification

Total fatty acid quantification was performed with a Free Fatty Acid Quantification Colorimetric/Fluorometric Kit (Biovision, Mountain View, CA, USA) according to the manufacturer’s instructions. Brain tissues were separated into hippocampus and cortex, and lysed. The protein concentration was measured using a BCA method. Samples were incubated with acetyl-CoA synthetase reagent, enzyme mix, and enhancer. Mixtures were incubated for 30 min and Total FA levels were detected using a microplate reader (at 550 nm).

### Lipid raft fractionation

Sucrose density gradient fractionation was performed to prepare flotillin- and caveolin-enriched membrane fractions, as described previously^79^. Cells were washed once with cold PBS and scraped into 2 ml of 500 mM sodium carbonate (pH 11.0), transferred to a plastic tube, and homogenized using a sonicator (three 20 sec bursts; Branson Sonicator 250, Branson Ultrasonic Corp., Danbury, CT, USA). The homogenate was then adjusted to 45% sucrose by the addition of 2 ml of 90% sucrose prepared in MES-buffered solution (MBS: 25 mM MES (pH 6.5), 0.15 M NaCl) and placed at the bottom of an ultracentrifuge tube. A 5–35% discontinuous sucrose gradient was formed above (4 ml of 5% sucrose, 4 ml of 35% sucrose, both in MBS containing 250 mM sodium carbonate) and centrifuged at 40,000 rpm for 20 h in an SW41 rotor (Beckman Instruments, CA, USA). Twelve samples of 1 ml fractions were collected and processed via western blot analysis.

### Immunocytochemistry

Cells were fixed with 20% acetone for 10 min at −20 °C, permeabilized with 0.01% Triton X-100/PBS for 5 min, and blocked in 5% normal goat serum (NGS) in PBS for 30 min. The cells were labeled with 5% NGS in PBS containing p-NF-κB, p-S6K1, p-mTOR, APP, and BACE1 antibodies at a ratio of 1:100, followed by fluorescein isothiocyanate-conjugated anti-rabbit and anti-mouse IgG antibody or Alexa Fluor goat anti-rabbit IgG counterstained with propidium iodide (PI, Life Technologies Gaithersburg, MD) for 1 h each at room temperature. Images were obtained using a FluoView^TM^ 300 confocal microscope (Olympus).

### Immunohistochemistry

Tissues were deparaffinized with xylene and 100, 95, 70, and 50% ethanol. For peroxide inactivation, tissues were then incubated on 3% H_2_O_2_ in methanol and washed twice with PBS. For antigen retrieval, tissue slides were incubated in pre-warmed (100 °C) citrate buffer (100 mM citrate, 0.05% Tween 20, pH 6.0 in D.W.) for 20 min and were washed twice with PBS for 5 min each. Subsequently, for permeabilization, tissues were incubated in PBS containing 0.5% Triton x100 for 15 min, washed three times with PBS, and incubated with 5% NGS in PBS for 30 min. The tissues were labeled with 5% NGS in PBS containing APP, BACE1, p-Tau and Aβ antibodies at a ratio of 1:100 for 2 h, followed by Alexa Fluor 488 secondary antibody (Life Technologies) and PI in 1:100 ratio for 1 h. After washing with PBS for 15 min, images were obtained using FluoView^TM^ 300 confocal microscope (Olympus). The percentage of threshold area was analyzed by using MetaMorph software (Universal Imaging, West Chester, PA, USA).

### Co-Immunoprecipitations

To isolate the native protein complexes from a lysate or secreted Aβ in medium by directly immobilizing purified antibodies onto an agarose support, we used co-immunoprecipitation kit (Thermo Fishers) according to the manufacturer’s instructions. Briefly, cells were lysed with cold IP lysis/wash buffer and incubated on ice for 5 min with periodic mixing. Cell debris was then removed by centrifugation (13,000 g, 10 min). Supernatants were transferred to determine the protein concentrations with BCA assays. CBP and Aβ antibodies were initially immobilized for 2 h with the AminoLink Plus coupling resin. The resin was then washed and incubated with the cell lysate or medium sample overnight. After incubation, the resin was washed again and the protein eluted using an elution buffer provided with kit. A negative control that was provided with the IP kit to assess the degree of nonspecific binding underwent treatment identical to that of the Co-IP samples. In this control, the coupling resin is not amine-reactive to prevent covalent immobilization of the primary antibody onto the resin. Samples were analyzed by western blot analysis.

### Statistical analysis

All data shown in the results are reported as a mean ± standard error of the mean (S.E.M). Data were compared using Student’s *t*-test for two-group analysis. Differences among experimental groups were assessed by using ANOVA. Comparisons of treatment groups with control groups were conducted by applying Bonferroni Dunn tests. A test with a *P*-value < 0.05 was considered significant.

## Electronic supplementary material

Supplementary information
