# Supplementary material for: Palmitic Acid-BSA enhances Amyloid-β production through GPR40-mediated dual pathways in neuronal cells: Involvement of the Akt/mTOR/HIF-1α and Akt/NF-κB pathways
Source: Sci Rep. 2017 Jun 28;7:4335. doi: 10.1038/s41598-017-04175-w (PMC5489526; doi:10.1038/s41598-017-04175-w)
Supplement: Supplementary file 1 — Supplementary information [file 41598_2017_4175_MOESM1_ESM.pdf]

Supplementary information

Palmitic Acid-BSA enhances Amyloid-β production through GPR40-mediated dual pathways in neuronal cells: Involvement of the Akt/mTOR/HIF-1α and Akt/NF-κB pathways

Jeong Yeon Kim[1, 2, #], Hyun Jik Lee[1, 2, #], Sei-Jung Lee[3, #], Young Hyun Jung[1, 2], Dae Young Yoo[2, 4], In Koo Hwang[2, 4, 5], Je Kyung Seong[2, 4, 5], Jung Min Ryu[6], and Ho Jae Han[1, 2, *]

[1]Department of Veterinary Physiology, College of Veterinary Medicine, Research Institute for Veterinary Science, Seoul National University, Seoul 08826, Republic of Korea.

[2]BK21 PLUS Program for Creative Veterinary Science Research Center, Seoul National University, Seoul 08826, Republic of Korea.

[3]Department of Pharmaceutical Engineering, Daegu Haany University, Gyeongsan 38610, Republic of Korea.

[4]Department of Anatomy and Cell Biology, College of Veterinary Medicine, Research Institute for Veterinary Science, Seoul National University,Seoul 08826, Republic of Korea.

[5]Korea Mouse Phenotyping Center (KMPC), Seoul 08826, Republic of Korea.

[6]Department of Veterinary Physiology, College of Veterinary Medicine, Chonnam National University, Gwangju 61186, Korea.

[*]Corresponding author: Ho Jae Han, D.V.M., Ph.D.

Department of Veterinary Physiology, College of Veterinary Medicine, Seoul National University, Gwanak-ro 1, Gwanak-gu, Seoul, 08826, South Korea

E-mail: hjhan@snu.ac.kr; Tel: 82-2-880-1261; Fax: 82-2-885-2732

[#] The first three authors contributed equally to this work.

Keywords

Alzheimer’s disease; palmitic acid; amyloid-β; HIF-1α; NF-κB

Supplementary figure legends

Supplementary Figure S1. Irrelevance of GPR120 in PA-BSA-induced Aβ production.

(a) mRNA expressions of *GPR40, GPR120 and ACTB* were showed with PCR analysis. (b). SK-N-MC cells were transfected with *GPR120* and NT siRNAs prior to incubation with PA-BSA (50 μM). Protein expression levels of APP, C99 and BACE1 were assessed with western blotting. Data are reported as a mean ± S.E.M. $n = 4$. Each blot result shown is representative image. *$p < 0.05$ versus control.

Supplementary Figure S2. Effect of PA-BSA on intracellular calcium release, ROS production, PKC phosphorylation.

(a) SK-N-MC cells were treated with PA-BSA (50 μM), and released calcium level was assessed using Fluo3AM staining. (b) SK-N-MC cells were incubated with 50μM of PA-BSA for 24 h, and ROS production level was analyzed with DCF-DA staining. Data are presented as a mean ± S.E.M. $n = 6$. (c) SK-N-MC cells were incubated with PA-BSA (50 μM) for 0-48 h. The phosphorylation of pan PKC was assessed with western blot. Data are presented as a mean ± S.E.M. $n = 3$. Each blot result shown is representative image. N.S means not significant.

Supplementary Figure S3. Role of GPR40 receptor signaling activated by PA-BSA in the phosphorylation of p70S6K1 and NF-κB.

(a-c) SK-N-MC cells were pre-treated with GW1100 (10 μM) for 30 min prior to for 24 h. p-GSK3β (Ser9), p-p70S6K1 (Thr389), p70S6K1, p-NF-κB p65 (Ser536), NF-κB p65 and β-actin were analyzed by western blot. Data are reported as a mean a mean ± S.E.M. $n = 4$. Each blot result shown is representative image. *$p < 0.05$ versus control, $^{\#}p < 0.05$ versus PA-BSA treatment.

Supplementary Figure S4. Effect of *HIF1A* siRNA transfection on HIF-1α expression induced by PA-BSA.

SK-N-MC cells were transfected with *HIF1A* and NT siRNAs for 12 h prior to incubation of PA-BSA

(50 μM) for 24 h. The expressions of HIF-1α and β-actin were analyzed by western blot. Data are reported as a mean ± S.E.M. $n = 3$. *$p < 0.05$ versus control, $^{\#}p < 0.05$ versus PA-BSA treatment.

Supplementary Figure S5. Role of Akt activated by PA-BSA induced-GSK3β phosphorylation.

SK-N-MCs were pre-treated with Akt inhibitor (1 μM) for 30 min prior to PA-BSA treatment (50 μM) for 24 h. Cells were blotted with p-GSK3β, p-Tau (Thr212), p-Tau (Ser396), Tau and β-actin. Data are presented as a mean ± S.E.M. $n = 3$. Each blot images are representative. . *$p < 0.05$ versus control, $^{\#}p < 0.05$ versus PA-BSA treatment.

Supplementary Figure S6. Effect of *APP* and *BACE1* siRNAs transfection on expressions of APP and BACE1.

(a) SK-N-MC cells were transfected with *APP* and NT siRNAs for 12 h prior to incubation of PA-BSA (50 μM) for 24 h. The expressions of APP and β-actin were analyzed by western blot. Data are reported as a mean ± S.E.M. $n = 4$. (b) SK-N-MC cells were transfected with *BACE1* and NT siRNAs for 12 h prior to treatment of PA-BSA (50 μM) for 24 h. The expressions of BACE1 and β-actin were assessed with western blot. Data are presented as a mean ± S.E.M. $n = 4$. Each blot result shown is representative image. *$p < 0.05$ versus control, $^{\#}p < 0.05$ versus PA-BSA treatment.

Supplementary Figure S7. Full-length western blot images of mice brain samples in the figure 1b.

All western blot images are full-length blot images of all mice brain samples in the figure 1b. Dash line box indicates cropped blot image in the figure 1b.

Supplementary Figure S8. Full-length western blot images for brain samples in the figure 1e.

All western blot images are full-length blot images of all mice brain samples in the figure 1e. Dash line box indicates cropped blot image in the figure 1e.

Supplementary Figure S9. Full-length western blot images for key data in the figure 2.

All western blot images are full-length blot images of key blot data in the figures 2. Dash line box indicates cropped blot image in the figure 2.

Supplementary Figure S10. Full-length western blot images for key data in the figure 3.

All western blot images are full-length blot images of key blot data in the figure 3. Dash line box indicates cropped blot image in the figure 3.

Supplementary Figure S11. Full-length western blot images for key data in the figure 4.

All western blot images are full-length blot images of key blot data in the figure 4. Dash line box indicates cropped blot image in the figure 4.

Supplementary Figure S12. Full-length western blot images for key data in the figure 5.

All western blot images are full-length blot images of key blot data in the figure 5. Dash line box indicates cropped blot image in the figure 5.

Supplementary Figure S13. Full-length western blot images for key data in the figure 6.

All western blot images are full-length blot images of key blot data in the figure 6. Dash line box indicates cropped blot image in the figure 6.

Supplementary Figure S14. Full-length western blot images for key data in the figure 7.

All western blot images are full-length blot images of key blot data in the figure 7. Dash line box indicates cropped blot image in the figure 7.

Supplementary figures

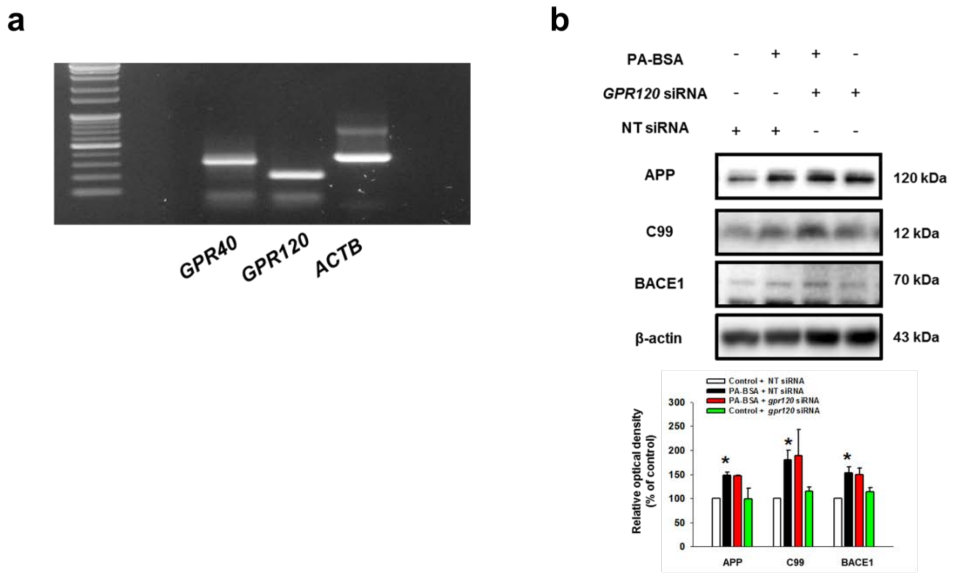

Sup. Fig. S1

.

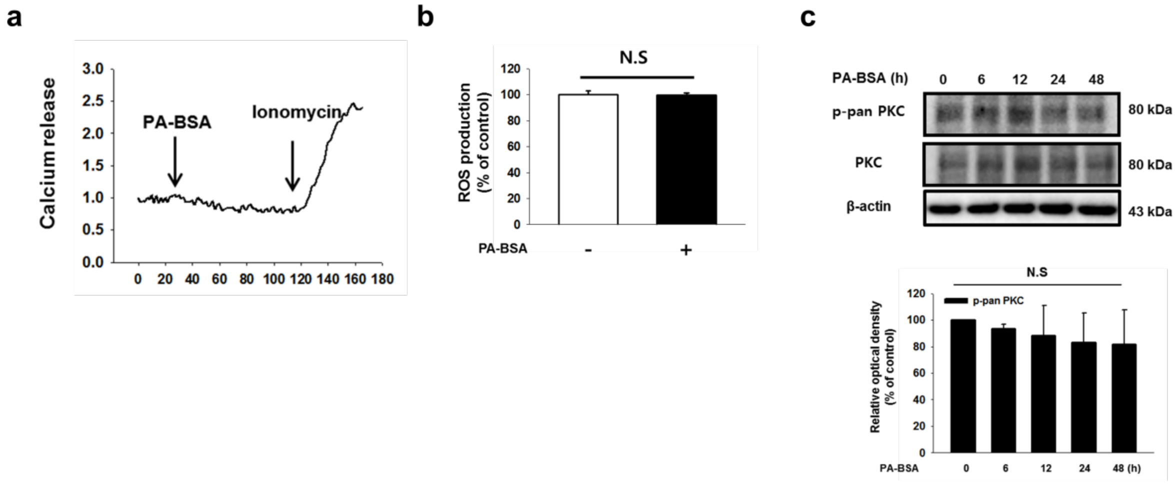
a
Calcium release
PA-BSA
Ionomycin
b
N.S
ROS production (% of control)
PA-BSA
-
+
c
PA-BSA (h)
0
6
12
24
48
p-pan PKC
PKC
β-actin
80 kDa
80 kDa
43 kDa
N.S
p-pan PKC
Relative optical density (% of control)
PA-BSA
48 (h)

Sup. Fig. S2

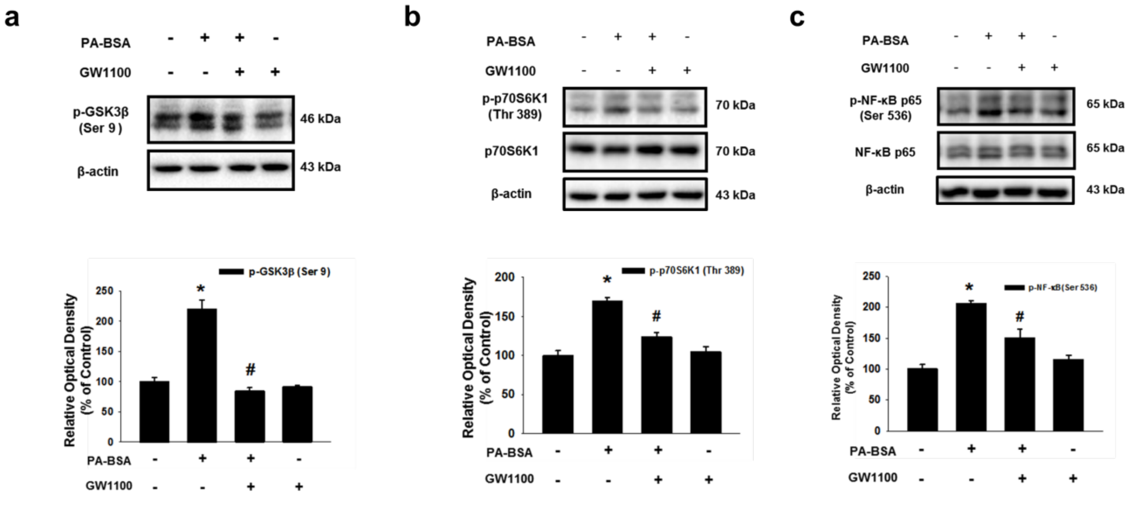

a
PA-BSA - + + -
GW1100 - - + +
p-GSK3β (Ser 9)
46 kDa
β-actin
43 kDa
p-GSK3β (Ser 9)
Relative Optical Density (% of Control)
0
50
100
150
200
250
*
#
PA-BSA - + + -
GW1100 - - + +
b
PA-BSA - + + -
GW1100 - - + +
p-p70S6K1 (Thr 389)
70 kDa
p70S6K1
70 kDa
β-actin
43 kDa
p-p70S6K1 (Thr 389)
Relative Optical Density (% of Control)
0
50
100
150
200
*
#
PA-BSA - + + -
GW1100 - - + +
c
PA-BSA - + + -
GW1100 - - + +
p-NF-κB p65 (Ser 536)
65 kDa
NF-κB p65
65 kDa
β-actin
43 kDa
p-NF-κB (Ser 536)
Relative Optical Density (% of Control)
0
50
100
150
200
250
*
#
PA-BSA - + + -
GW1100 - - + +

Sup. Fig. S3

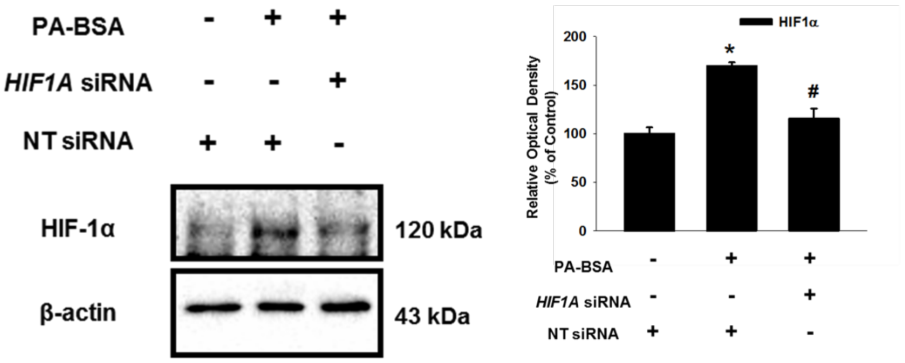
PA-BSA
HIF1A siRNA
NT siRNA
HIF-1α
β-actin
120 kDa
43 kDa
HIF1α
Relative Optical Density (% of Control)
200
150
100
50
0
*
#
PA-BSA
HIF1A siRNA
NT siRNA

Sup. Fig. S4

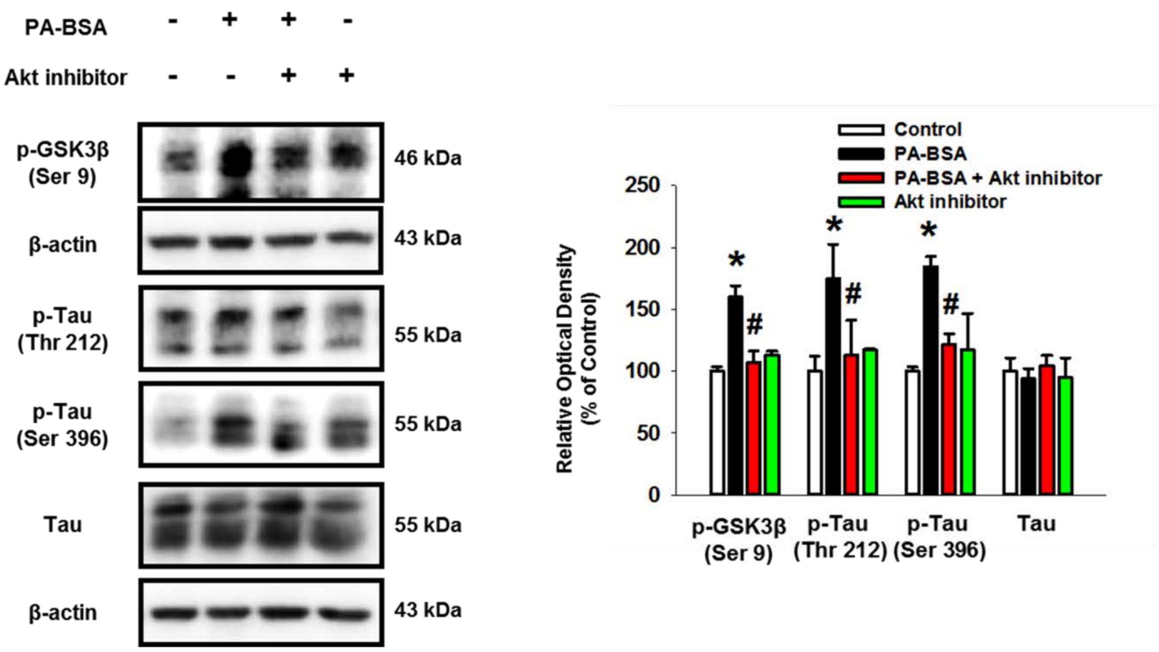
PA-BSA - + + -
Akt inhibitor - - + +
p-GSK3β (Ser 9)
46 kDa
β-actin
43 kDa
p-Tau (Thr 212)
55 kDa
p-Tau (Ser 396)
55 kDa
Tau
55 kDa
β-actin
43 kDa
Control
PA-BSA
PA-BSA + Akt inhibitor
Akt inhibitor
Relative Optical Density (% of Control)
250
200
150
100
50
0
p-GSK3β (Ser 9)
p-Tau (Thr 212)
p-Tau (Ser 396)
Tau

Sup. Fig. S5

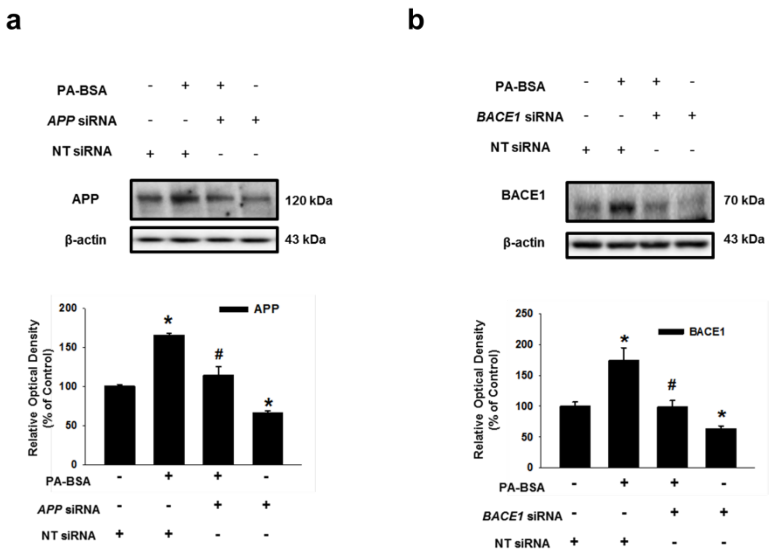
a
PA-BSA
APP siRNA
NT siRNA
APP
120 kDa
β-actin
43 kDa
Relative Optical Density (% of Control)
APP
b
PA-BSA
BACE1 siRNA
NT siRNA
BACE1
70 kDa
β-actin
43 kDa
Relative Optical Density (% of Control)
BACE1

Sup. Fig. S6

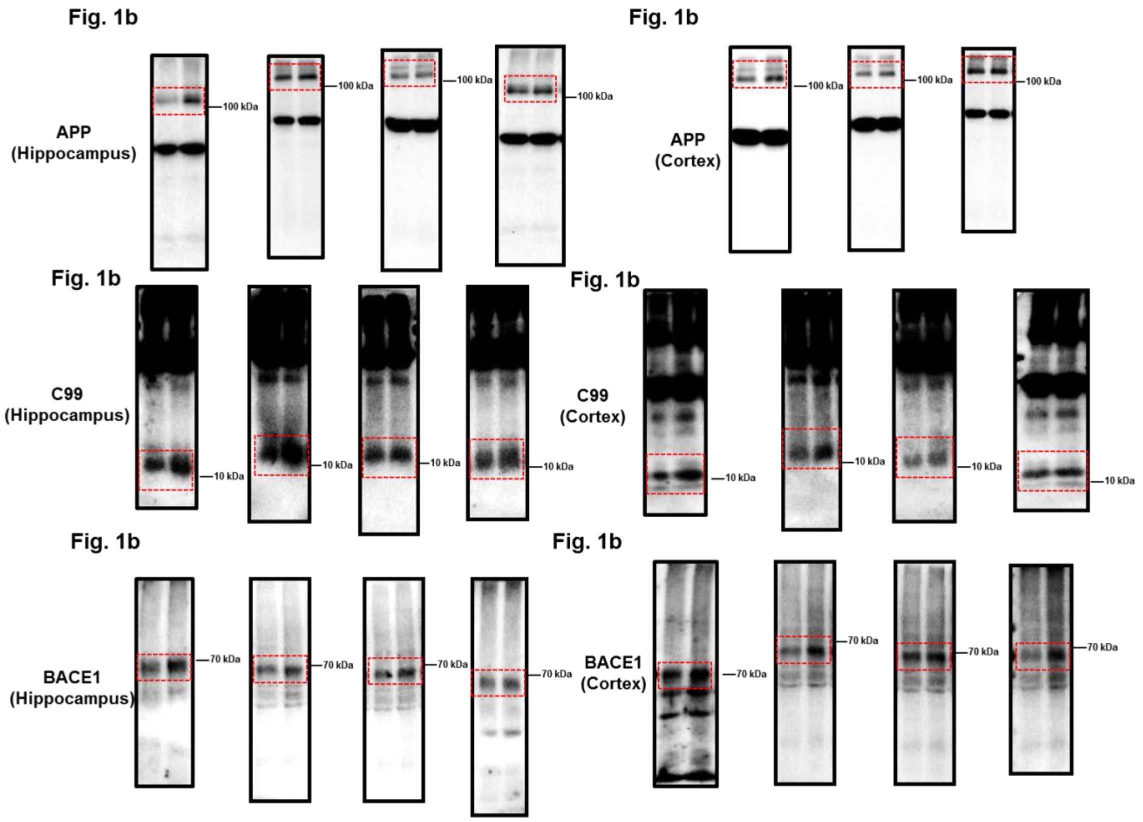
Fig. 1b
APP
(Hippocampus)
100 kDa
Fig. 1b
APP
(Cortex)
100 kDa
Fig. 1b
C99
(Hippocampus)
10 kDa
Fig. 1b
C99
(Cortex)
10 kDa
Fig. 1b
BACE1
(Hippocampus)
70 kDa
Fig. 1b
BACE1
(Cortex)
70 kDa

Sup. Fig. S7

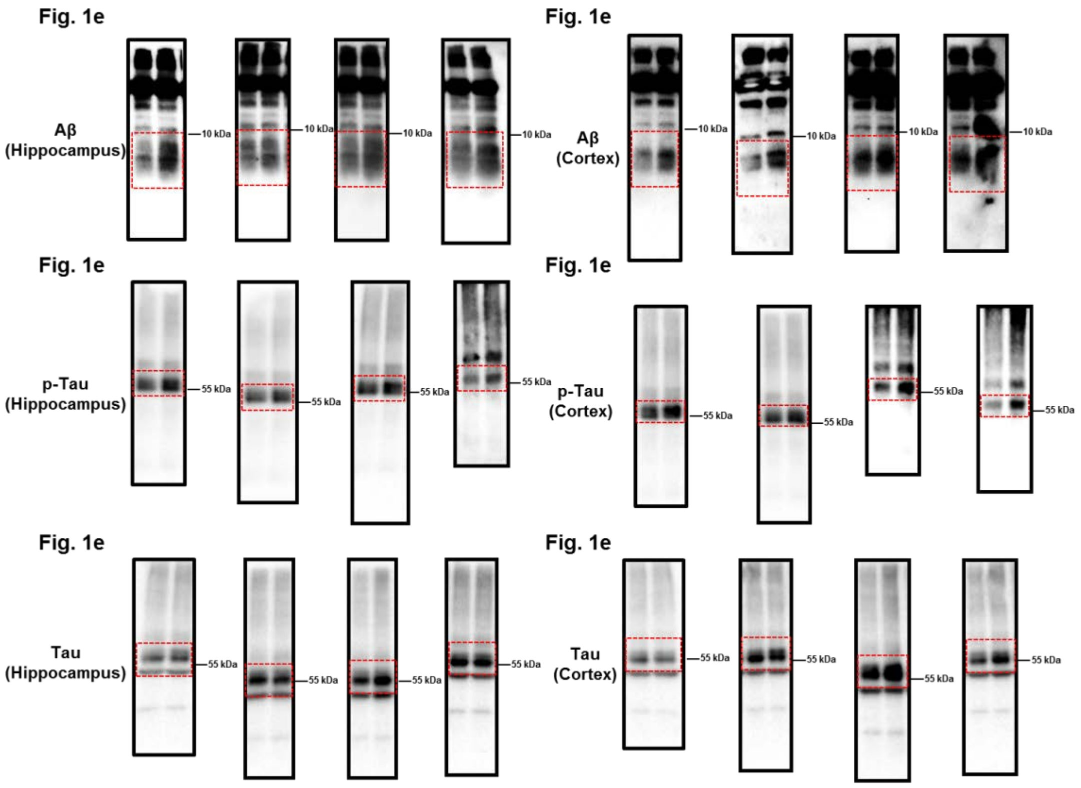
Fig. 1e
Aβ (Hippocampus)
10 kDa
Fig. 1e
Aβ (Cortex)
10 kDa
Fig. 1e
p-Tau (Hippocampus)
55 kDa
Fig. 1e
p-Tau (Cortex)
55 kDa
Fig. 1e
Tau (Hippocampus)
55 kDa
Fig. 1e
Tau (Cortex)
55 kDa

Sup. Fig. S8

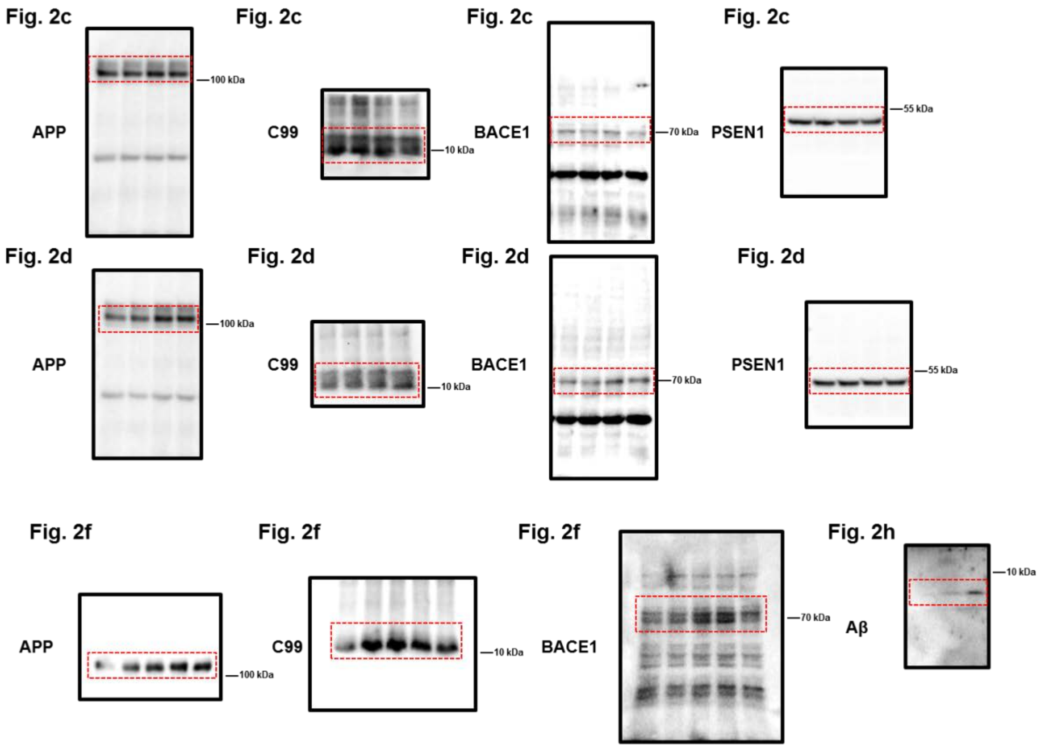
Fig. 2c
APP
100 kDa
Fig. 2c
C99
10 kDa
Fig. 2c
BACE1
70 kDa
Fig. 2c
PSEN1
55 kDa
Fig. 2d
APP
100 kDa
Fig. 2d
C99
10 kDa
Fig. 2d
BACE1
70 kDa
Fig. 2d
PSEN1
55 kDa
Fig. 2f
APP
100 kDa
Fig. 2f
C99
10 kDa
Fig. 2f
BACE1
70 kDa
Fig. 2h
Aβ
10 kDa

Sup. Fig. S9

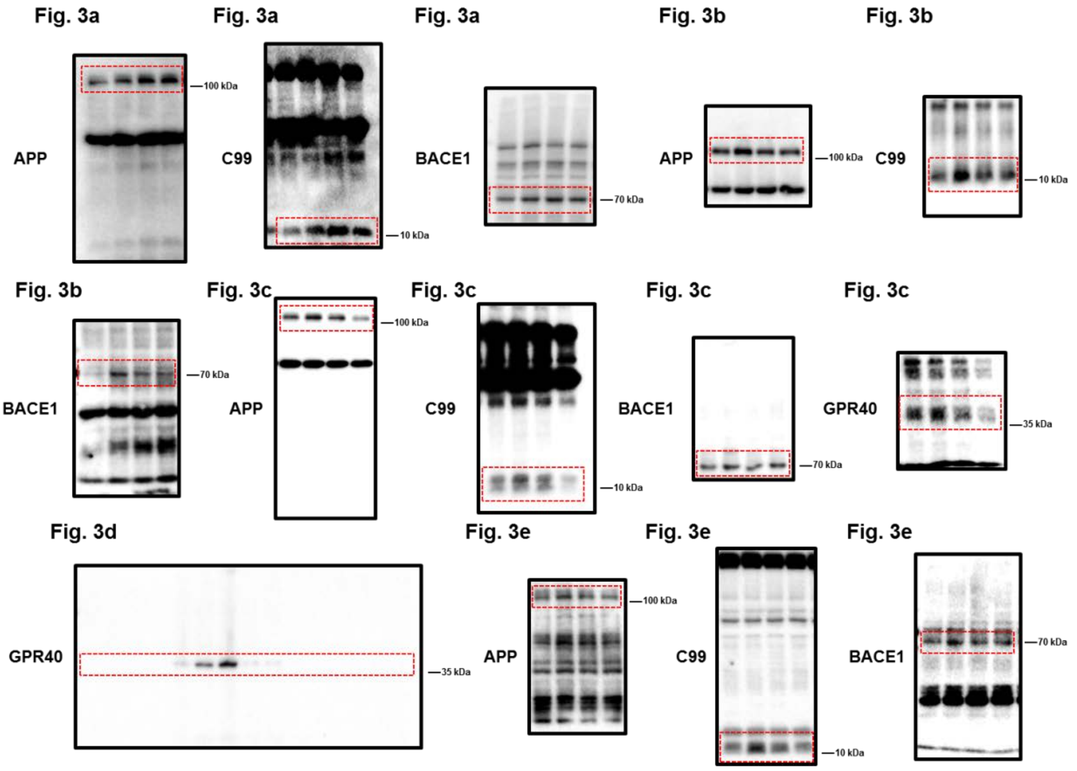
Fig. 3a
APP
100 kDa
Fig. 3a
C99
10 kDa
Fig. 3a
BACE1
70 kDa
Fig. 3b
APP
100 kDa
Fig. 3b
C99
10 kDa
Fig. 3b
BACE1
70 kDa
Fig. 3c
APP
100 kDa
Fig. 3c
C99
10 kDa
Fig. 3c
BACE1
70 kDa
Fig. 3c
GPR40
35 kDa
Fig. 3d
GPR40
35 kDa
Fig. 3e
APP
100 kDa
Fig. 3e
C99
10 kDa
Fig. 3e
BACE1
70 kDa

Sup. Fig. S10

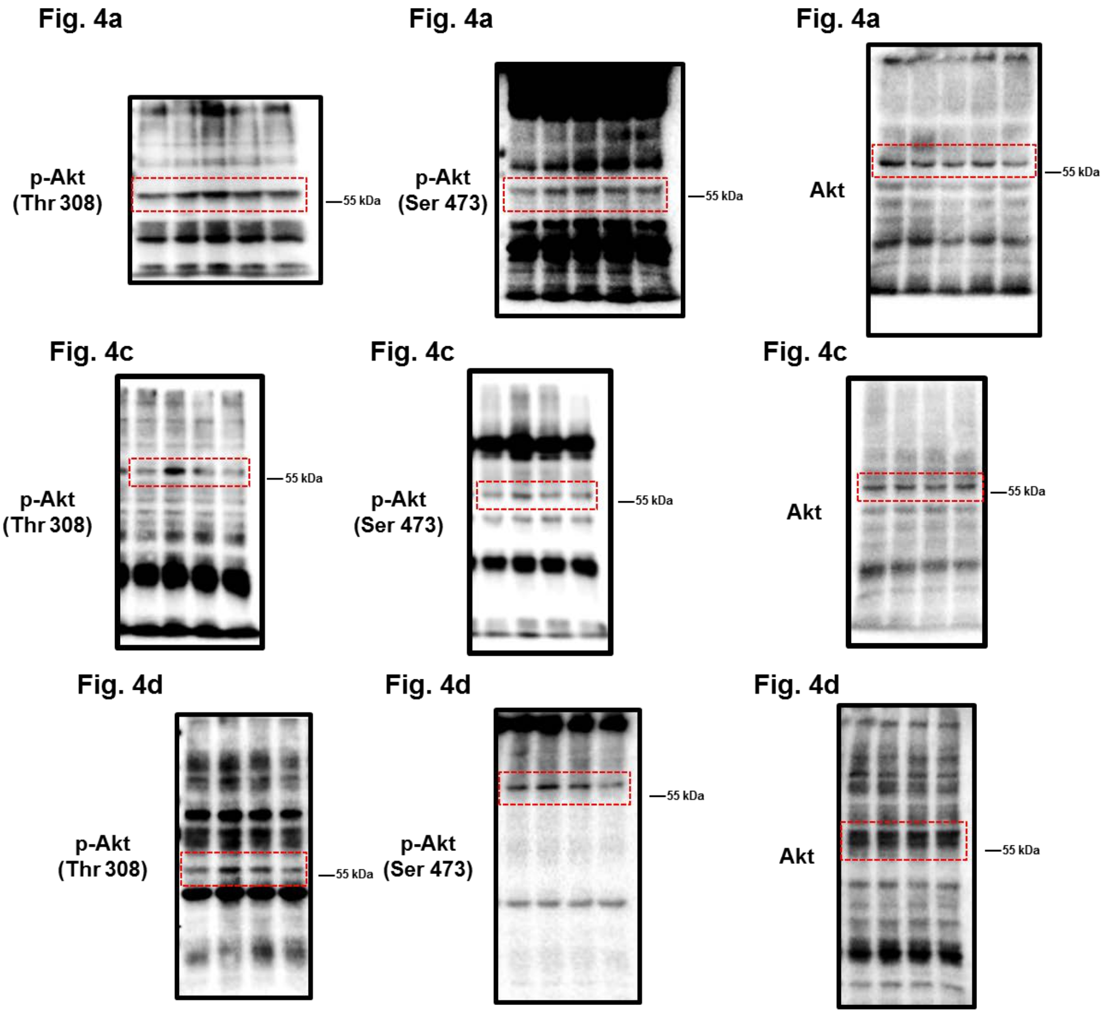
Fig. 4a
p-Akt
(Thr 308)
55 kDa
Fig. 4a
p-Akt
(Ser 473)
55 kDa
Fig. 4a
Akt
55 kDa
Fig. 4c
p-Akt
(Thr 308)
55 kDa
Fig. 4c
p-Akt
(Ser 473)
55 kDa
Fig. 4c
Akt
55 kDa
Fig. 4d
p-Akt
(Thr 308)
55 kDa
Fig. 4d
p-Akt
(Ser 473)
55 kDa
Fig. 4d
Akt
55 kDa

Sup. Fig. S11

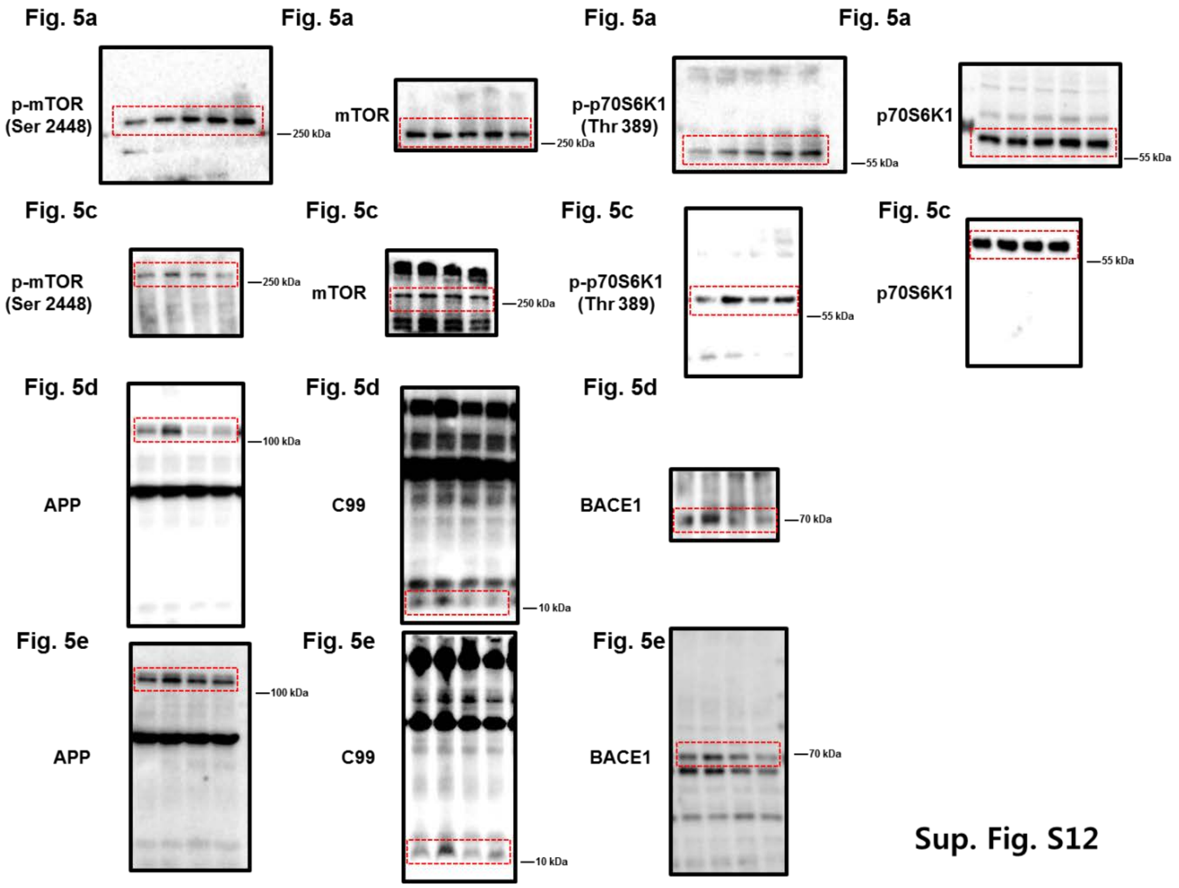
Fig. 5a
p-mTOR (Ser 2448)
250 kDa
Fig. 5a
mTOR
250 kDa
Fig. 5a
p-p70S6K1 (Thr 389)
55 kDa
Fig. 5a
p70S6K1
55 kDa
Fig. 5c
p-mTOR (Ser 2448)
250 kDa
Fig. 5c
mTOR
250 kDa
Fig. 5c
p-p70S6K1 (Thr 389)
55 kDa
Fig. 5c
p70S6K1
55 kDa
Fig. 5d
APP
100 kDa
Fig. 5d
C99
10 kDa
Fig. 5d
BACE1
70 kDa
Fig. 5e
APP
100 kDa
Fig. 5e
C99
10 kDa
Fig. 5e
BACE1
70 kDa

Sup. Fig. S12

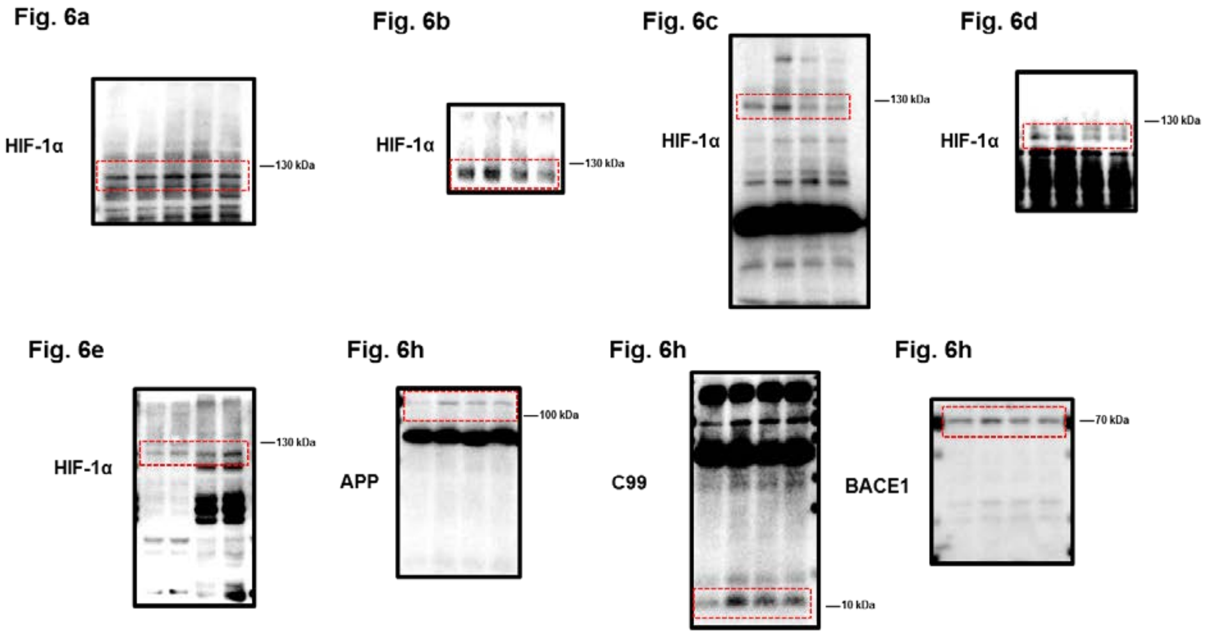
Fig. 6a
HIF-1α
130 kDa
Fig. 6b
HIF-1α
130 kDa
Fig. 6c
HIF-1α
130 kDa
Fig. 6d
HIF-1α
130 kDa
Fig. 6e
HIF-1α
130 kDa
Fig. 6h
APP
100 kDa
Fig. 6h
C99
10 kDa
Fig. 6h
BACE1
70 kDa

Sup. Fig. S13

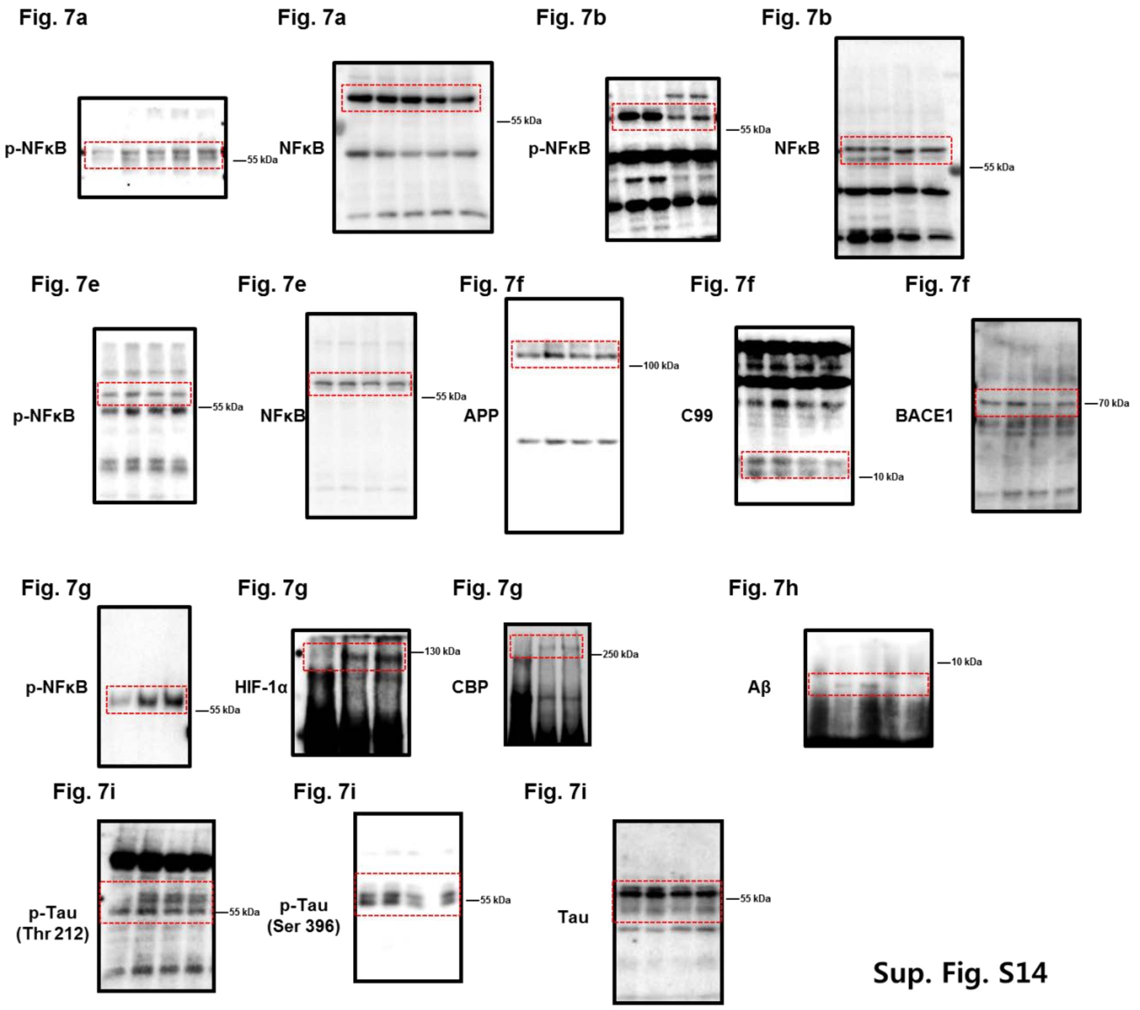
Fig. 7a
p-NFκB
55 kDa
Fig. 7a
NFκB
55 kDa
Fig. 7b
p-NFκB
55 kDa
Fig. 7b
NFκB
55 kDa
Fig. 7e
p-NFκB
55 kDa
Fig. 7e
NFκB
55 kDa
Fig. 7f
APP
100 kDa
Fig. 7f
C99
10 kDa
Fig. 7f
BACE1
70 kDa
Fig. 7g
p-NFκB
55 kDa
Fig. 7g
HIF-1α
130 kDa
Fig. 7g
CBP
250 kDa
Fig. 7h
Aβ
10 kDa
Fig. 7i
p-Tau
(Thr 212)
55 kDa
Fig. 7i
p-Tau
(Ser 396)
55 kDa
Fig. 7i
Tau
55 kDa

Sup. Fig. S14

Supplementary Table S1. Sequences of primers used for RT-PCR and real-time PCR

Gene name	Species	Identification	Sequence (5'-3')	Size (bp)
GPR40	Human	Sense	CTGGTCTACGCCCTGAACCT	316
		Antisense	GAGCCTCCAACCCAAAGACC	
GPR120	Human	Sense	CTTCTTCTCCGACGTCAAGG	201
		Antisense	GGGATAGCGCTGATGAAGAG	
ACTB	Human	Sense	AACCGCGAGAAGATGACC	351
		Antisense	AGCAGCCGTGGCCATCTC	
APP	Human, Mouse	Sense	TAGAAGTAGCAGAGGAGGAAG	244
		Antisense	TACCAGCGGGAGATCATT	
BACE1	Human	Sense	CTGCCTGGATTTCTTCCTATTA	255
		Antisense	CTTGTGGTGGAGGACATAAG	
GAPDH	Human	Sense	GTCCACCACTGACACGTTG	156
		Antisense	GGGAAACTGTGGCGTGAT	
Bace1	Mouse	Sense	ACATATCGAGACCTCCGAAAGG	149
		Antisense	AACTTGTCCGATTCAGTGATGG	
Actb	Mouse	Sense	GCAGGAGTACGATGAGTCCG	239
		Antisense	ATCCTGAGTCAAAAGCGCCA	

Supplementary Table S2. Sequences of siRNAs used for gene silencing

Target gene	Sequences 5’-3’	Manufacturer
APP	GGUGGGCGGUGUUGUCAUA<br>GGUUCUGGGUUGACAAAUA<br>CGGAAACGAUGCUCUCAUG<br>CUAUUCAGAUGACGUCUUG	Dharmacon
BACE1	GCAAGGAGUACAACUAUGA<br>GGAGGGAGCAUGAUCAUUG<br>UAUGGGAGCUGUUAUCAUG<br>AGACGACUGUUACAAGUUU	Dharmacon
GPR40	CGCUCAACGUCCUGGCCAU<br>GUGACCGGUUACUUGGGAA	Dharmacon
GPR120	GAAAUGACUUGUCGAUUAU<br>CAAGAGCUGUCGUGACUCA<br>GGACUGGUCAUUGUGAUCA<br>GGAAGAGGCUCACGGUAAG	Dharmacon
HIF1A	GCCGCUCAAUUUAUGAAUATT<br>UAUUCAUAAAUUGAGCGGCTT	Dharmacon
Non-targeting	UAGCGACUAAACACAUCAA<br>UUGAUGUGUUUAGUCGCUA	Dharmacon

Supplementary Table S3. Sequences of CHIP primers used for RT-PCR and real-time PCR

Gene	Identification	Sequence (5'-3')	Size (bp)
APP promoter for HIF-1α	Forward	ACTCTCCCTCCCACTGTTCA	178
	Reverse	CACCCGAGAGAGACCCCTAG	
BACE1 promoter for HIF-1α	Forward	ACCACCTTCTCCCACTGAGT	161
	Reverse	CAGGCGCTGGAGATACAGAG	
APP promoter for NF-κB	Forward	ACTCTCCCTCCCACTGTTCA	178
	Reverse	CACCCGAGAGAGACCCCTAG	
BACE1 promoter for NF-κB	Forward	CTGACAGACGGGAGGTGTG	174
	Reverse	GGTTTTCGCTTTTCCCTGGG	
